# Identification of Potential SARS-CoV-2 Main Protease (MPro) Inhibitors Through Pharmacophore Modeling, Molecular Docking, and Molecular Dynamics Simulation Approaches

**DOI:** 10.3390/ijms27177684

**Published:** 2026-08-27

**Authors:** Mohd Yasir Khan, Farah Maarfi, Abid Ullah Shah, Nithyadevi Duraisamy, Mohammed Cherkaoui, Maged Gomaa Hemida

**Affiliations:** 1 Department of Digital Engineering and Artificial Intelligence, College of Science, Long Island University, Brooklyn, NY 11201, USA; mohd.yasirkhan@liu.edu (M.Y.K.); farah.maarfi@liu.edu (F.M.); nithyadevi.duraisamy@liu.edu (N.D.); mohammed.cherkaoui@liu.edu (M.C.); 2 Department of Veterinary Biomedical Sciences, Lewyt College of Veterinary Medicine, Long Island University, 720 Northern Boulevard, Brookville, NY 11548, USA; abidullah.shah@liu.edu

**Keywords:** coronaviruses (CoVs), main protease (MPro), pharmacophore modeling, virtual screening, molecular docking, molecular dynamics simulation (MDS), catalytic dyad

## Abstract

The main protease (MPro) of coronaviruses (CoVs) is an essential enzyme involved in viral replication and represents an attractive target for antiviral drug discovery. Based on the similar binding pocket residues within the MPro of different CoVs, this study aimed to identify potential inhibitors of SARS-CoV-2 MPro from PDB ID 6M2N using integrated computational approaches. Interaction-based pharmacophore modeling, virtual screening, molecular docking, MM-GBSA binding energy calculation, and molecular dynamics simulation (MDS) were performed using BIOVIA Discovery Studio. The validated pharmacophore model was utilized to screen the ZINC database, followed by docking and 100 ns MDS analyses of the top-ranked compounds. The pharmacophore model 01 demonstrated favorable predictive performance (AUC = 0.781). Virtual screening identified 483 compounds, from which 15 compounds were selected for docking studies. Among them, ZINC95473654 (Lig-1), ZINC95473725 (Lig-2), and ZINC08792368 (Lig-3) exhibited strong binding affinity toward MPro. Lig-1 demonstrated the best docking score and binding free energy, along with stable interactions with key catalytic residues HIS41, CYS145, and GLU166. MDS analyses further confirmed that Lig-1, Lig-2 and Lig-3 maintained stable conformations. The hydrogen bond distance monitoring and post MDS-MM-GBSA results suggest Lig-1 followed by Lig-3 as an inhibitor for MPro and persistent intermolecular interactions throughout the 100 ns simulation period. The findings suggest that Lig-1, followed by Lig-3, may serve as promising computational lead compounds targeting SARS-CoV-2 MPro, representing promising candidates for further experimental validation.

## 1. Introduction

Coronaviruses (CoVs) are a diverse group of enveloped, (+) sense RNA viruses that infect a wide range of mammalian and avian hosts, causing respiratory, enteric, hepatic, and neurological diseases [1,2,3,4,5]. They are taxonomically classified into four genera (*Alphacoronavirus*, *Betacoronavirus*, *Gammacoronavirus*, and *Deltacoronavirus*) based on the phylogenetic and genomic characteristics [1,2,3,4,5]. The *Betacoronaviruses* include some important human coronaviruses and are classified into three subgenera including Sarbeco, Merbeco, and Embeco coronaviruses. The emergence of highly pathogenic Sarbecocoroanviruses, including SARS-CoV in 2002, MERS-CoV in 2012, and SARS-CoV-2 in 2019, has underscored the global health threat posed by zoonotic spillover events and highlighted the urgent need for some potent and effective antiviral therapeutics [2,6,7,8,9]. One of the emerging approaches in vaccine and drug designs is to monitor the genomic makeup of the pathogens to ensure that the developed vaccines and drugs are targeting the currently circulating strains of viruses infecting humans, animals and birds [6,8,10,11,12,13,14,15,16,17,18,19,20]. The genome structure and organization of CoVs are highly conserved across different groups, particularly within the 5′ two-thirds of the genome, which encodes the nonstructural proteins (NSPs). These NSPs are encoded as a large polyprotein that consists of two overlapping open reading frames (ORFs) with a ribosomal frameshifting in between. These ORFs are cleaved into 16 non-structural proteins (NSPs). There are two viral encoded proteases that cleave the long polypeptide into 16 NSPs. The NSp3 encodes the papain-like protease (PLp), while the main viral protease is encoded by the NSP5 called 3C Like protease or the main protease (MPro). The full-length genomes of CoVs are flanked by two untranslated regions (UTRs) at both ends. A central feature shared across all known coronaviruses is the presence of a highly conserved main protease (MPro), a cysteine protease indispensable for viral replication. MPro is responsible for the proteolytic cleavage of the large viral polyproteins pp1a and pp1ab into several functional non-structural proteins that are essential for viral replication, transcription, and assembly [21]. Because MPro is somewhat conserved among human and animal coronaviruses and lacks closely related homologs in mammalian host cells, it has emerged as one of the most attractive antiviral targets for the development of some effective coronavirus therapeutics within a One Health framework. The conservation of the catalytic dyad composed of HIS41 and CYS145 across diverse coronaviruses further supports the feasibility of identifying inhibitors that may target conserved catalytic residues across coronavirus MPro enzymes [22].

Computational drug discovery has emerged as a powerful strategy for accelerating antiviral development. Structure-based pharmacophore modeling, virtual screening, molecular docking, andMDS enable rapid identification of compounds with favorable binding characteristics prior to experimental validation. Based on the similar binding pocket residues within the MPro of different CoVs, the study aimed to identify some potential inhibitors using integrated computational approaches. These in silico methods are particularly valuable for conserved viral targets like MPro, where subtle differences in binding pocket architecture can be exploited to design inhibitors with strong antiviral potential [23,24,25].

Among the important amino acids of the MPro binding pocket, HIS41 and CYS145 belong to the catalytic dyad of MPro, which has been reported to play an important role in inhibiting SARS-CoV-2 replication. This catalytic dyad (HIS41–CYS145) is responsible for cleaving the viral polyproteins pp1a and pp1ab at more than 11 conserved sites to release non-structural proteins (NSPs) essential for assembly of the replication transcription. Because humans lack closely related homologs of this protease, MPro is considered one of the most attractive antiviral drug targets for CoVs [26,27].

Structural studies have demonstrated that MPro is a homodimeric enzyme whose activity depends on proper dimerization and the integrity of key residues such as HIS41, CYS145, and GLU166; the latter stabilizes the S1 substrate binding pocket and supports dimer formation. Consistent with this, our study shows that the amino acid (Glu166) plays an important role in the dimerization of the MPros. The multiple sequence alignment of the MPro from representative α- and β-coronaviruses in our study revealed that HIS41, CYS145, and GLU166 are highly conserved, further supporting their importance and the potential of MPro as a target for the development of broad-spectrum coronavirus inhibitors [28].

The therapeutic relevance of MPro is further supported by the success of protease inhibitors in other RNA viruses, including HIV and HCV. For SARS-CoV-2 specifically, several small-molecule inhibitors such as nirmatrelvir, the active component of Paxlovid, have demonstrated clinical efficacy by directly targeting MPro. However, the rapid evolution of CoVs in both humans and animals, combined with the ongoing risk of zoonotic transmission, necessitates the discovery of some cross-species MPro inhibitors that align with a One Health approach [29].

Among the most important veterinary coronavirus protease inhibitors is GC376, which was initially developed against feline infectious peritonitis virus (FIPV), a highly fatal feline coronavirus affecting domestic and wild cats. GC376 demonstrated potent inhibition of FIPV replication both in vitro and in naturally infected cats through direct targeting of the viral MPro enzyme. Treatment with GC376 resulted in significant health improvement and prolonged survival in cats with naturally occurring feline infectious peritonitis (FIP). Importantly, subsequent structural and biochemical studies revealed that GC376 also effectively inhibits the MPro enzymes of SARS-CoV, MERS-CoV, and SARS-CoV-2, highlighting their potential antiviral activities. Because feline coronaviruses share substantial structural similarities with human coronaviruses, GC376 became one of the earliest examples illustrating how antiviral discoveries in veterinary medicine can directly contribute to human pandemic preparedness [30,31]. In this study, we employed a comprehensive computational pipeline including pharmacophore modeling, ZINC database virtual screening, molecular docking, binding energy estimation, and 100 ns MDS to identify novel small molecule inhibitors capable of targeting conserved catalytic residues of MPro from SARS-CoV-2. Our findings highlight promising compounds, particularly ZINC95473654 (Lig-1) and ZINC08792368 (Lig-3), which demonstrated strong binding affinities, stable interactions with key active site residues HIS41, CYS145, and GLU166, and favorable dynamic stability during MDS. Post-MDS binding-energy analysis further supported the stability of these protein–ligand complexes. These compounds therefore warrant further biochemical and antiviral evaluation. Given the conservation of key catalytic and binding-site residues across several coronaviruses, future experimental studies should investigate whether these compounds exhibit inhibitory activity against MPro enzymes from other coronaviruses [31].

## 2. Results

### 2.1. Multiple Sequence Alignment Using MPro from Representative Coronaviruses

**Figure 1:**
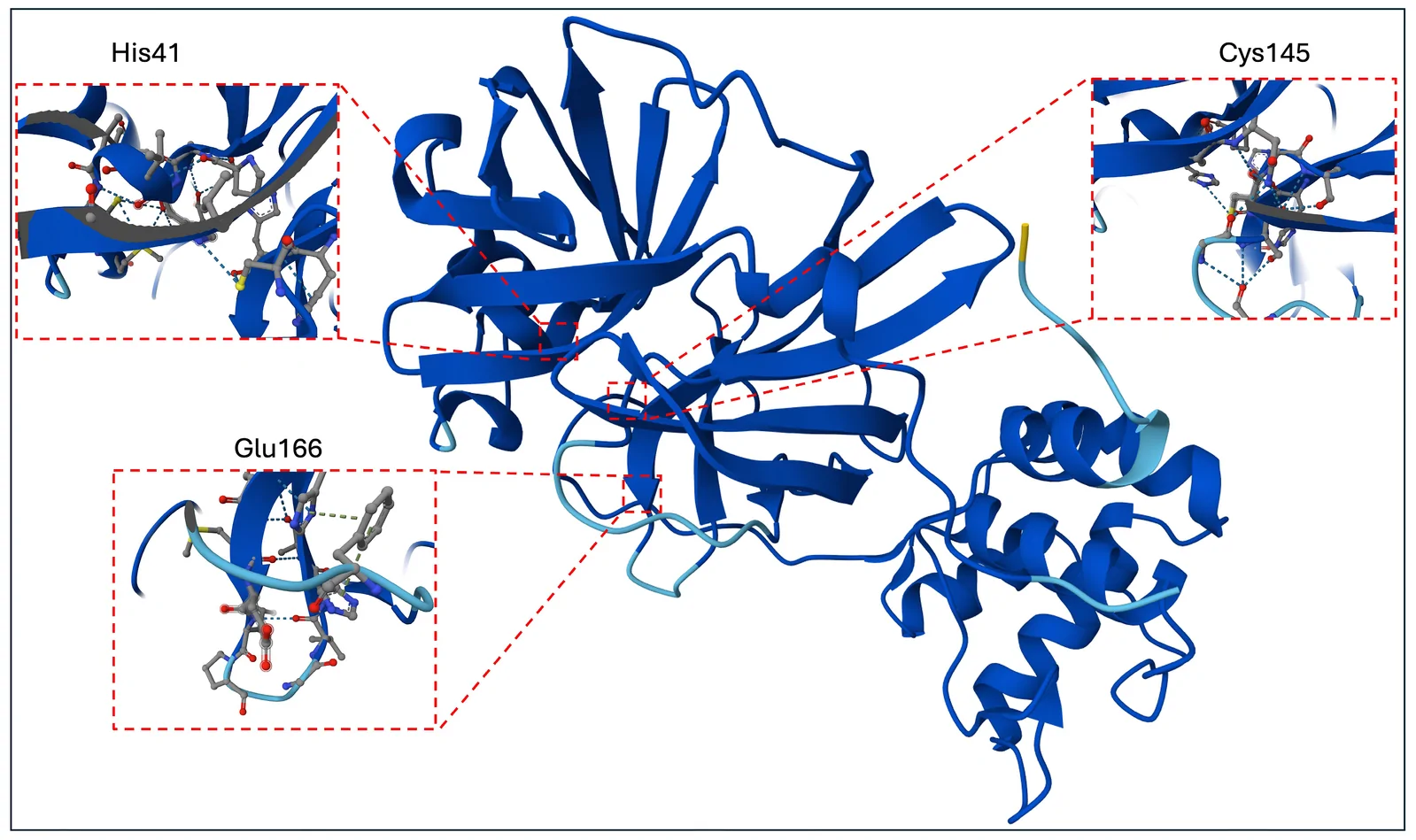
3D structure of SARS-CoV-2 MPro (PDB ID: 6M2N). The protein structure is shown in ribbon representation, with the highlighted active-site regions enlarged in the inset panels. The catalytic dyad residues HIS41 and CYS145 and the binding-pocket residue GLU166 are shown in stick representation. Red dashed boxes and connecting lines indicate the locations of the enlarged residue views. Protein colors represent the structural ribbon representation.

Among the important amino acids of the MPro binding pocket, HIS41 and CYS145 belong to the catalytic dyad of MPro, which has been reported to play an important role in inhibiting SARS-CoV-2 replication (Figure 1) [29,32,33]. The Glu166 plays an important role in the dimerization of MPro [34,35].

**Figure 2:**
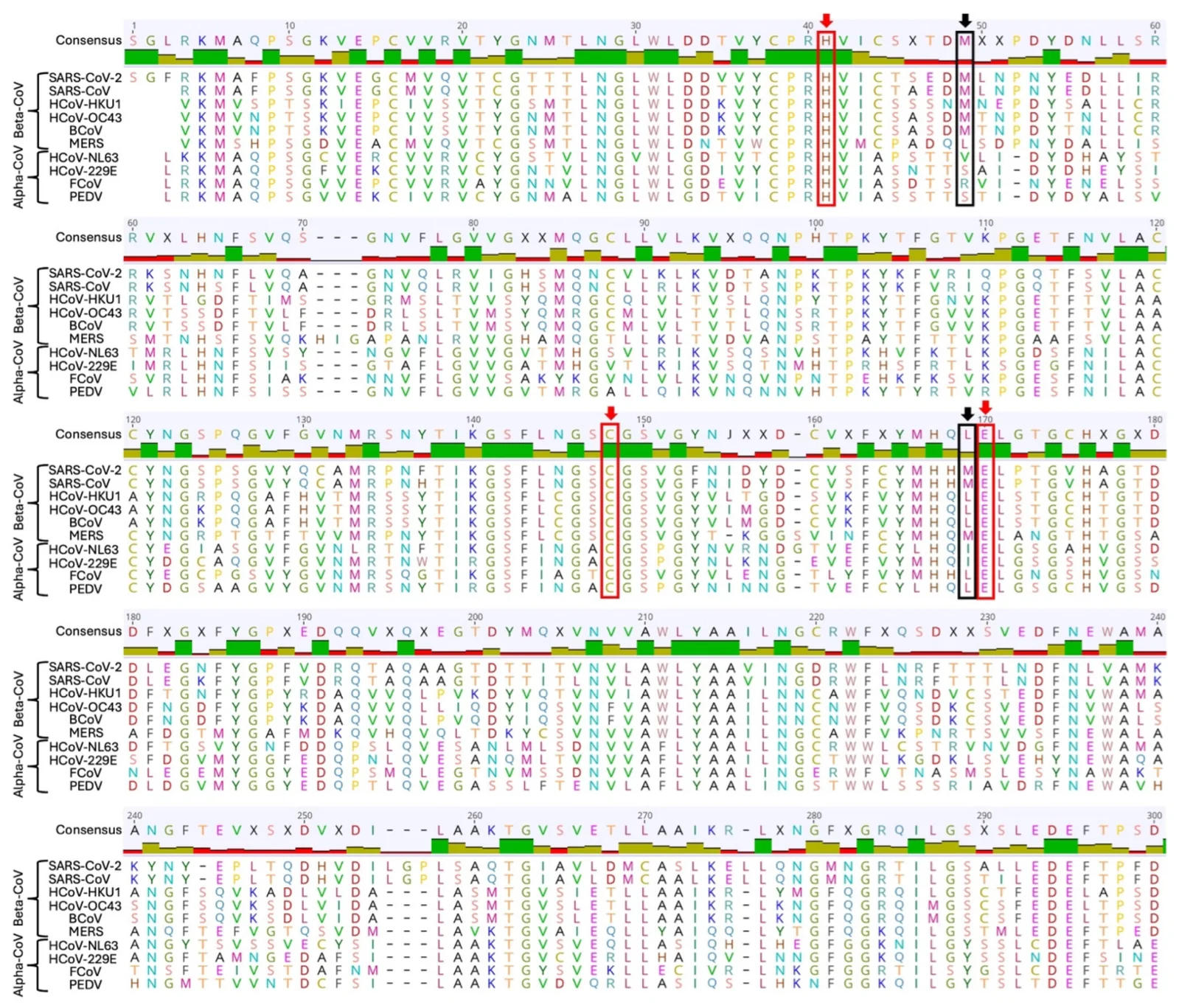
Multiple sequence alignment of the MPro protein of different *Alphacoronaviruses* and *Betacoronaviruses*. MPro of six *Betacoronaviruses* and four *Alphacoronaviruses* were used to perform multiple sequence alignment. Among the five important amino acids interacting with compounds, three amino acids, which are conserved among all ten coronaviruses, are indicated with a red box and red arrowhead. Two amino acids that are not conserved are shown with a black box and black arrowhead. The multiple sequence alignment was performed using Geneious Prime V.9.0.

To highlight these residues of the binding pocket of MPro, we performed multiple sequence alignment using MPro from 10 different coronaviruses. Among these 10 coronaviruses, four coronaviruses including HCoV-NL63, HCoV-229E, FCoV, and PEDV belong to the *Alphacoronvirus* group, while six coronaviruses including SARS-CoV-2, SARS-CoV, HCoV-HKU1, HCoV-OC43, BCoV, and MERS belong to *Betacoronaviruses*. Results showed that HIS41, CYS145, and GLU166 amino acids are conserved among all ten coronaviruses. However, Met49 was only conserved among the coronaviruses of the *Betacoronvirus* family except MERS (Figure 2).

### 2.2. Pharmacophore Modeling Validation and Virtual Screening

Interaction-based pharmacophore generation using the co-crystallized SARS-CoV-2 MPro–ligand complex produced ten candidate pharmacophore models composed of different combinations of hydrogen bond acceptor (A), hydrophobic aromatic (Harom), and aromatic ring (R) features. The models were ranked according to their predicted selectivity, resulting in four or five pharmacophoric features together with receptor-derived excluded volume constraints. These constraints represent sterically inaccessible regions within the binding cavity of proteins, and thereby improve the structural selectivity of virtual screening [36,37].

The generated pharmacophore models were validated using a dataset comprising 38 experimentally active compounds and 39 inactive compounds. The predictive ability of each model was assessed by receiver operating characteristic (ROC) analysis, and the corresponding sensitivity, specificity, true positives, true negatives, false positives, false negatives, and areas under the ROC curves (AUCs) were evaluated. Among the ten generated pharmacophore models, Pharmacophore model 1 (PPM-1) exhibited the best overall validation performance and was selected for subsequent pharmacophore-based virtual screening depicted in Table 1.

**Figure 3:**
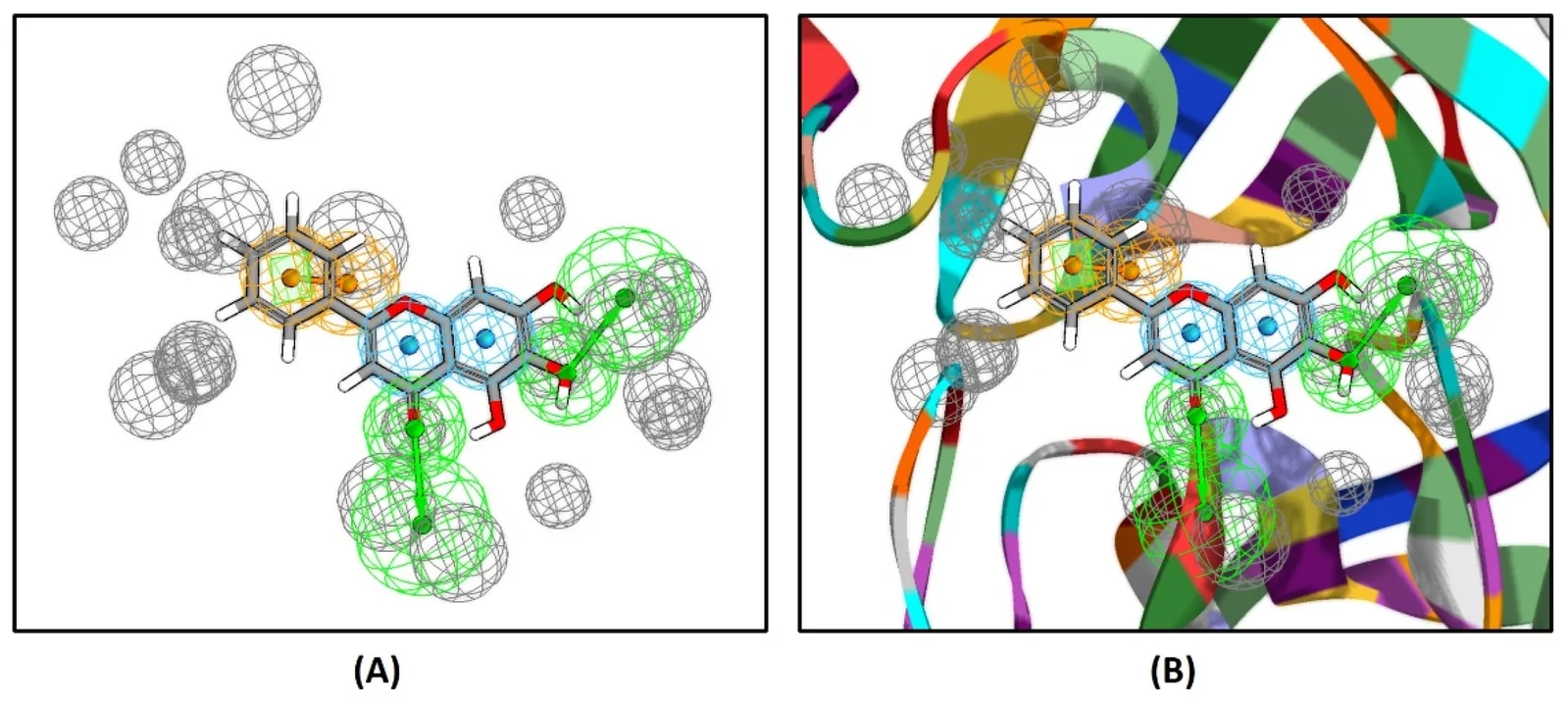
Interaction-based pharmacophore model. (**A**) Three-dimensional representation of the selected Pharmacophore_01 (AAHaromHaromR) highlighting the essential pharmacophoric features, including hydrogen bond acceptors (blue), hydrophobic aromatic features (green), aromatic ring feature (yellow), and receptor-derived excluded volume constraints (gray). (**B**) Spatial orientation of the validated pharmacophore model within the SARS-CoV-2 MPro ligand-binding cavity, illustrating the correspondence between the pharmacophore features and the experimentally defined receptor ligand interaction environment used for subsequent pharmacophore-based virtual screening.

The selected PPM-1 corresponds to a five-feature AAHaromHaromR pharmacophore comprising two hydrogen bond acceptor features, two hydrophobic aromatic features, and one aromatic ring feature, together with 19 receptor-based excluded volume constraints that define the steric architecture of the ligand-binding pocket (Figure 3). These excluded volumes represent sterically inaccessible regions within the binding cavity, thereby improving the selectivity of virtual screening.

Validation against a dataset of 77 compounds (38 active and 39 inactive compounds) demonstrated that PPM-1 correctly identified 31 active compounds (true positives) and correctly rejected 24 inactive compounds (true negatives), yielding a sensitivity of 0.81579, specificity of 0.61538, and an AUC-ROC value of 0.781 (Table 1). Although the specificity indicates a moderate false-positive rate, the model provides a balanced compromise between recovering active compounds and limiting the exclusion of potential true positives, making it suitable for pharmacophore-based virtual screening. In contrast, PPM-3, PPM-4, PPM-5, and PPM-7 exhibited very high sensitivities (>0.94) but extremely poor specificities (<0.21), resulting in substantially higher false-positive rates (Table 1) and reduced screening efficiency. Therefore, despite identifying more active compounds, these models were not considered suitable for subsequent virtual screening.

The selected AAHaromHaromR PPM-1 architecture is also consistent with the experimentally reported binding mechanism of non-covalent SARS-CoV-2 MPro inhibitors, where ligand recognition is mediated by a combination of hydrogen bonding, aromatic, and hydrophobic interactions within the catalytic pocket [38]. Accordingly, the two hydrogen bond acceptor features capture essential polar interactions, whereas the two hydrophobic aromatic features and one aromatic ring feature represent the π–π stacking and hydrophobic contacts that stabilize ligand binding. Based on these validation results, PPM-1 was employed as the query model for pharmacophore-based virtual screening using the ZINCPharmer platform, which enables pharmacophore-based screening of commercially available compounds from the ZINC database (https://zincpharmer.csb.pitt.edu/, accessed on 13 December 2025). This filtering step yielded 483 hit compounds that were subsequently subjected to structural ligand preparation, duplicate removal, bad valencies fixes and energy minimization filters, to 230 compounds. Out of 483 compounds, 253 compounds were removed due to duplicate structures, failed structure preparation, invalid valencies, or unsuccessful energy minimization After removal of duplicates, the remaining 230 compounds were then again mapped onto the validated PPM-1 model through internal mapping with a pharmacophore mapping method in BIOVIA discovery studio, and their compatibility was assessed using the pharmacophore fit value. Consequently, 49 compounds with a fit value range from 3.5 to 0.02 were mapped, although 21 compounds out of these 49 compounds with fit values ranging from 3.5 to 1.0 exhibited acceptable geometric and chemical complementarity with validated pharmacophore models. Based on this, 28 compounds were excluded because their pharmacophore fit values were <1.0 with the validated pharmacophore model. Therefore, 21 compounds falling within this fit value range (3.5–1) were selected for subsequent downstream virtual screening analyses (Table S1).

### 2.3. Drug-Likeness Evaluation and ADMET Profiling of the Screened Compounds

The 21 compounds identified through pharmacophore-based virtual screening were initially evaluated for drug-likeness using Lipinski’s Rule of Five and Veber’s rule, together with PAINS screening. Based on their overall drug-likeness profiles and absence of PAINS alerts, 15 of the 21 compounds were prioritized for further analysis. These criteria were used as guidelines for compound prioritization rather than as strict exclusion rules. Most shortlisted compounds showed overall physicochemical profiles consistent with the evaluated drug-likeness criteria, although individual compounds exhibited one or more deviations. Several compounds exhibited ALogP values slightly above the Lipinski guideline of 5, indicating relatively high lipophilicity; however, these compounds were retained based on their overall physicochemical profiles rather than excluded on the basis of a single parameter. Similarly, ZINC08792368, despite marginally exceeding the Lipinski molecular-weight guideline (507.537 Da), satisfied the remaining Lipinski and Veber parameters and showed no PAINS alert and was therefore retained for downstream analysis (Table 2). The chemical identifiers of these compounds are given in Tables S1 and S2.

These 15 compounds were subsequently subjected to ADMET prediction using the BIOVIA Discovery Studio ADMET module to evaluate their pharmacokinetic and toxicity profiles (Table 3).

The assessed parameters included aqueous solubility, human intestinal absorption, blood–brain barrier (BBB) penetration, CYP2D6 inhibition, hepatotoxicity, and plasma protein binding (PPB). The majority of the shortlisted compounds exhibited very low to low aqueous solubility (solubility levels 1–3), which is considered acceptable for many orally active drug candidates, together with good to low intestinal absorption (absorption levels 0–2), indicating favorable oral absorption characteristics for most compounds. All selected compounds were predicted to be non-inhibitors of CYP2D6 (FALSE), suggesting a lower potential for CYP2D6-mediated drug–drug interactions. Furthermore, several compounds were predicted to be non-hepatotoxic, while others showed a hepatotoxicity signal and should therefore be interpreted with caution during subsequent lead optimization. Most compounds were also predicted to exhibit high plasma protein binding (PPB ≥ 90%), which may prolong systemic circulation but could reduce the free pharmacologically active fraction. In addition, the majority of the compounds showed BBB level 4 predictions, indicating very low or undefined blood–brain barrier penetration, which may be advantageous for a non-central nervous system antiviral target such as SARS-CoV-2 MPro by minimizing potential central nervous system exposure. Overall, the ADMET analysis identified compounds with favorable pharmacokinetic profiles that were subsequently prioritized for molecular docking and molecular dynamics simulations.

### 2.4. Redocking Validation for the Molecular Docking Process

**Figure 4:**
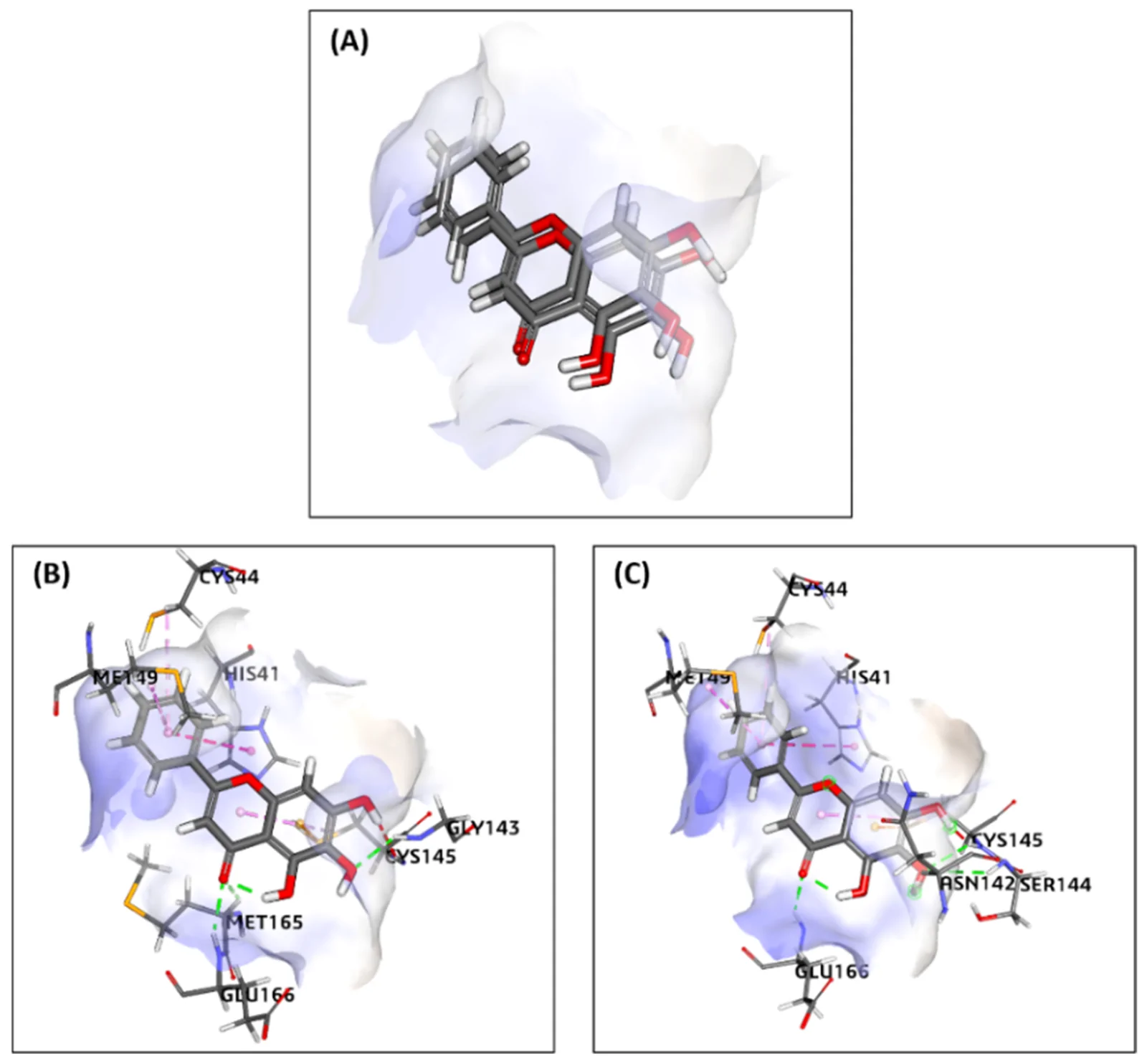
Redocking validation of the co-crystallized Baicalein inhibitor with MPro protein (PDB ID: 6M2N). (**A**) Superimposition of the redocked and native co-crystallized Baicalein within the active-site pocket. (**B**) Interaction profile of the native Baicalein–MPro complex showing interactions with HIS41, MET49, GLY143, CYS145, MET165, and GLU166 residues. (**C**) Interaction profile of the redocked Baicalein–MPro complex demonstrating conserved interactions with HIS41, MET49, SER144, CYS145, ASN142, and GLU166 active-site residues.

To further validate docking results of selected ligands from the virtual screening method, the MPro receptor protein was re-docked with native Baicalein (standard ligand). The redocking’s binding site area was x: −33.218228, y: −65.348922, z: 41.208347, with a 6.85 sphere radius. The parameter of the validation method was RMSD. The RMSD analysis showed the degree of deviation from experimental ligand docking results to the native ligand (standard) attached with the MPro protein at the same binding site. The higher the RMSD value, the greater the deviation, which indicates the higher prediction error of ligand–protein interactions. Conversely, the low RMSD value is attributed to better conformation because the redocking ligand position is closer to the ligand position resulting from crystallography. The redocking results indicated a 0.683 Å RMSD value from the native ligand with the MPro receptor (Figure 4). Therefore, based on the low RMSD value between the crystallized native and re-docked ligand, the method used for redocking in this study is valid and can be used against tested ligands with the same binding site area.

### 2.5. MPro-Ligands Interaction and Binding Energy Calculations

**Figure 5:**
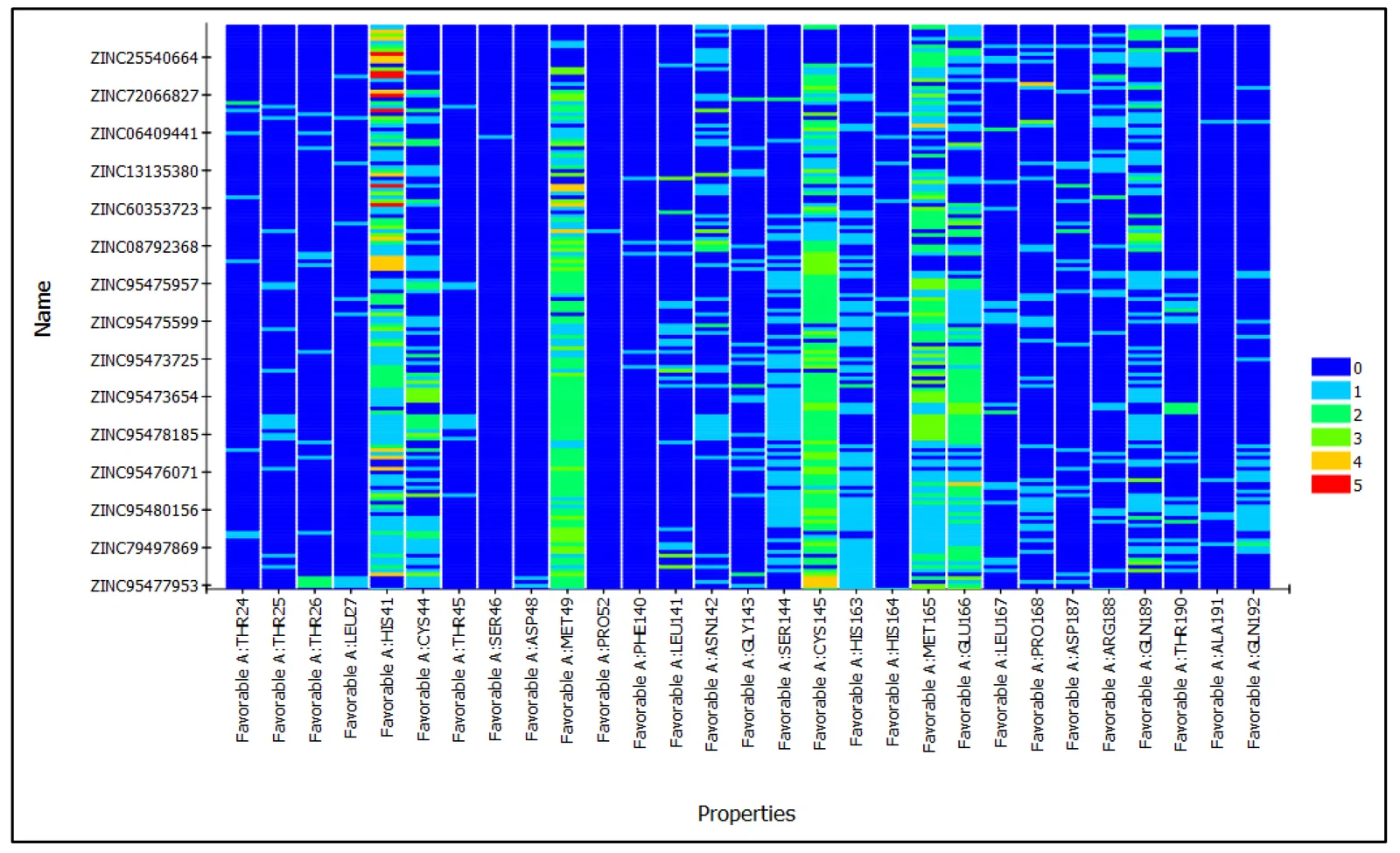
Heatmap exhibiting interaction patterns of selected 15 ZINC compounds with key binding-site residues. Rows represent ligands and columns represent residue-specific interactions. The color scale (blue to red) indicates interaction intensity, from low/no interaction (blue) to strong interaction (red). Key residues such as HIS41, CYS145, MET49, MET165, and GLU166 exhibit prominent interaction hotspots, highlighting their importance in ligand binding.

The MPro in complex with the inhibitor compound Baicalein in X-ray crystal structure from the RCSB PDB database (PDB ID: 6M2N; 2.20 Å resolution) was chosen as the receptor for different ligand docking. The residues included HIS41 and CYS145, which form an essential catalytic dyad in the structure of MPro. The interaction pattern of the top 10 poses of selected ligands from docking results clearly exhibited that most of the ligands interacted with catalytic dyad residues HIS41 and CYS145 and other important residues of the binding pocket of MPro (Table 4 and Figure 5).

In the attempt to identify compounds with the strongest binding affinity toward MPro, molecular docking analysis demonstrated favorable interactions for all 15 selected compounds, with −CDOCKER scores ranging from 53 to 28 kcal/mol and Pre-MDS-MM-GBSA binding energies ranging from −121 to −23 kcal/mol against the active-site residues of MPro (Table S3). To further validate the docking results using an independent docking engine, the top-ranked compounds and the reference ligand were additionally evaluated using AutoDock Vina (v1.1.2, Windows), which yielded consistent binding affinity trends.

**Figure 6:**
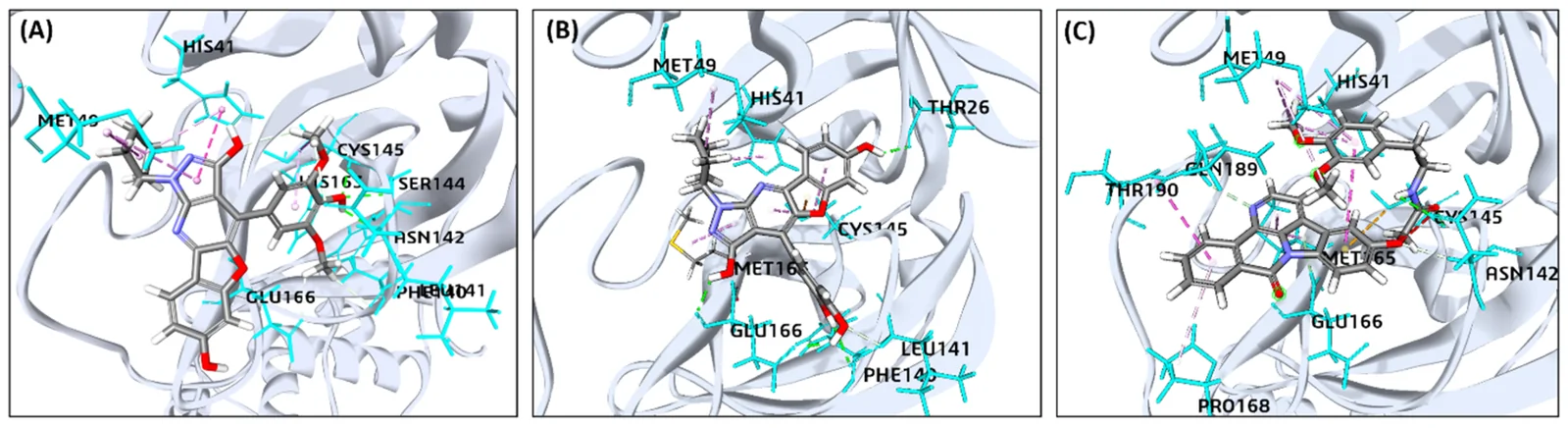
Protein–ligand interaction profiles of (**A**) Lig-1, (**B**) Lig-2, and (**C**) Lig-3 complexes within the SARS-CoV-2 MPro active-site cavity, showing interactions with key catalytic and binding pocket residues including HIS41, CYS145, GLU166, MET49, MET165, ASN142, SER144, THR190, and PRO168.

Among all screened compounds, ZINC95473654, ZINC95473725, and ZINC08792368 exhibited the highest docking affinities, with −CDOCKER scores of 54.60, 50.83, and 52.73 kcal/mol, respectively, and Pre-MDS MM-GBSA binding energies of −121.86, −102.47, and −97.73 kcal/mol, respectively. Independent validation using AutoDock Vina further supported these findings, with binding affinities of −8.7, −8.6, and −8.1 kcal/mol, respectively, all exceeding that of the reference ligand Baicalein (−7.5 kcal/mol). The comparable ranking obtained using BIOVIA, −CDOCKER and AutoDock Vina, together with the favorable predicted interaction profiles, provided complementary support for prioritizing these compounds for subsequent MDS (Table 5). In contrast, the reference ligand Baicalein exhibited a comparatively lower binding affinity (−CDOCKER score: 34.23 kcal/mol; Pre-MDS-MM-GBSA: −90.02 kcal/mol) and lower AutoDock Vina binding affinity (−7.5 kcal/mol), indicating weaker predicted binding than the top-ranked screened compounds (Figure 6 and Table 5).

The stability of the ZINC95473654–MPro complex is mediated by interactions with key active-site residues, including HIS41, MET49, PHE140, LEU141, CYS145, SER144, ASN142, HIS163, and GLU166. Conventional hydrogen bonds formed with SER144 and CYS145 play a critical role in anchoring the ligand within the binding pocket. In addition, π–π stacking interactions involving HIS41 and hydrophobic contacts with MET49 and LEU141 further contribute to ligand stabilization. Surrounding residues such as PHE140, ASN142, HIS163, and GLU166 provide additional support through van der Waals interactions, collectively ensuring a stable ligand–protein complex.

ZINC95473725 exhibited a predicted binding pose involving THR26, PHE140, GLU166, CYS145, HIS41, MET49, MET165, and LEU141. Conventional hydrogen-bond interactions were observed with THR26, PHE140, GLU166, and CYS145, while hydrophobic and π–alkyl interactions involving HIS41, MET49, and MET165 contributed to the predicted ligand orientation within the active site. Additional van der Waals contacts with LEU141 further complemented the ligand–binding-site interaction profile. Collectively, these interactions indicated favorable accommodation of ZINC95473725 within the MPro active-site cavity.

The residues involved in the ZINC08792368-MPro complex’s stability were HIS41, MET49, ASN142, CYS145, MET165, GLU166 and PRO168. The residue CYS145 and ASN142 were found to form strong hydrogen bond interactions, while PRO168 and MET-165 contributed to hydrophobic and π–π stacking interactions.

The interaction analysis revealed that the ligand forms conventional hydrogen bonds with residues CYS145 and ASN142, contributing to binding stability. Additional stabilization is provided by amide–π stacking interactions with PRO168 and hydrophobic contacts involving MET49, MET165, and HIS41. However, the absence of strong hydrogen bonding interactions with key catalytic residues such as HIS41 and GLU166 suggests comparatively weaker binding affinity relative to more optimal ligands. Weak interactions, including carbon hydrogen bonds and van der Waals contacts, further support ligand positioning within the binding pocket. The interaction patterns of the top three ligands ZINC95473654, ZINC95473725, ZINC08792368, and standard compound (Baicalein), are also given in 2D (Figure S2).

### 2.6. Stability of the Protein MPro–Ligand Complexes During MDS

**Figure 7:**
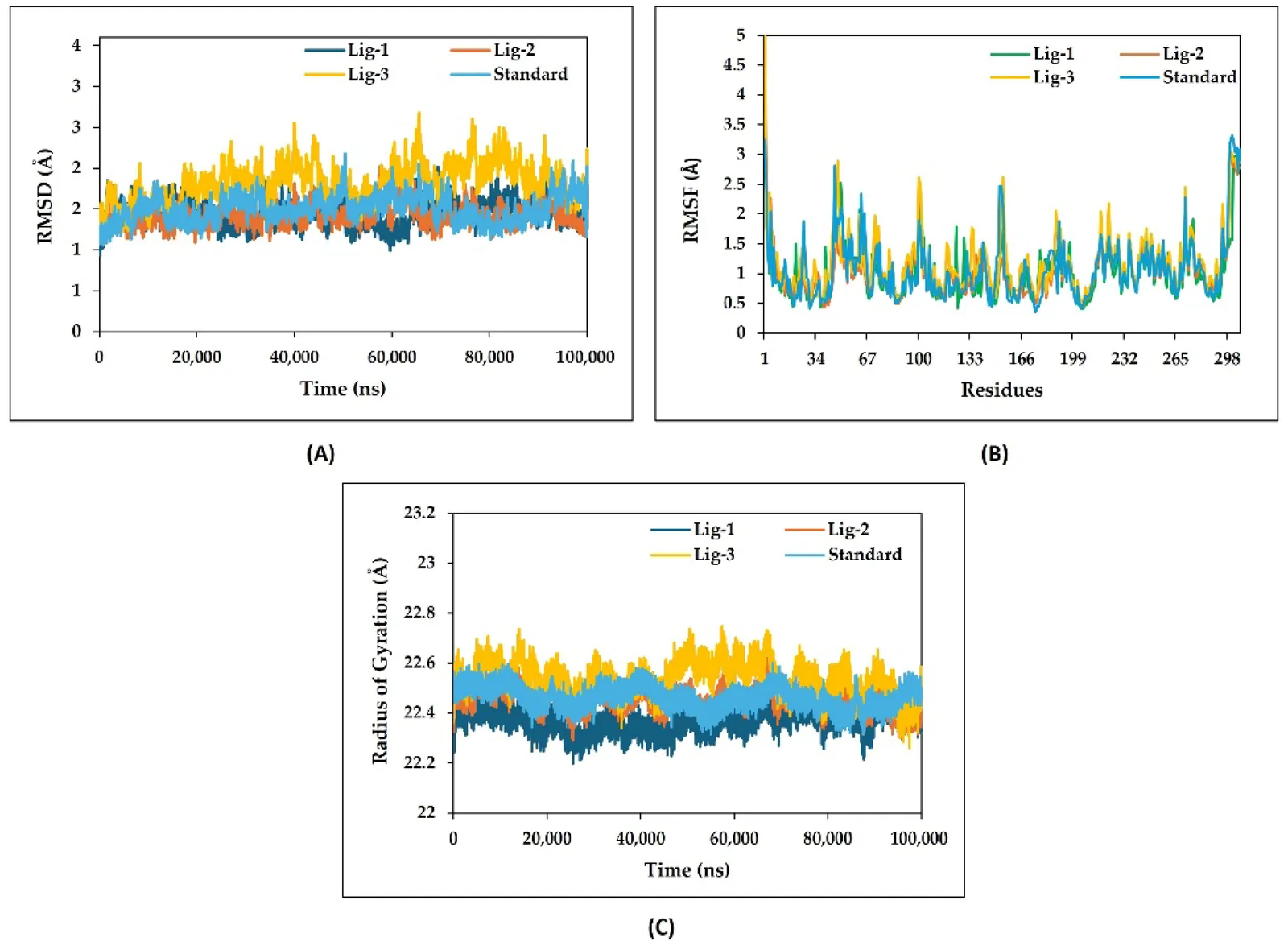
Molecular dynamics simulation analysis of the Standard, Lig-1, Lig-2, and Lig-3 complexes over 100 ns. (**A**) RMSD analysis showing structural stability of the complexes during the simulation period. (**B**) RMSF analysis representing residue-wise flexibility of the protein complexes. (**C**) Radius of gyration (RG) analysis indicating the compactness and conformational stability of the complexes throughout the simulation trajectory.

RMSD analysis was carried out over a 100 ns molecular dynamics simulation to evaluate the structural stability of the MPro protein in complex with compound ZINC95473654 as Ligand-1 (Lig-1), ZINC95473725 as Ligand-2 (Lig-2), and ZINC08792368 as Ligand-3 (Lig-3), in comparison with the standard (Baicalein–MPro) complex. As shown in Figure 7A, all ligands–MPro complex systems exhibited an initial equilibrated phase during the early stage of the simulation and remained relatively stable throughout the remaining simulation period. The RMSD, RMSF, and RG profiles shown represent the average of two independent MDS.

The Lig-1 complex demonstrated the highest structural stability, maintaining an average RMSD between approximately 1.2 and 1.6 Å, with only minor fluctuations across the simulation trajectory. Similarly, the Lig-2 complex exhibited a stable RMSD profile ranging from nearly 1.2 to 1.7 Å, indicating stable binding and minimal conformational deviation of the protein–ligand complex. The standard complex also maintained a comparatively stable trajectory, with RMSD values fluctuating around 1.3–1.9 Å during the 100 ns simulation. Among all three selected ligands, the Lig-3 complex displayed relatively higher fluctuations, particularly after 30 ns, where RMSD values increased to approximately 2.0–2.5 Å with occasional transient peaks. Despite these fluctuations, the complex remained within an acceptable RMSD range, suggesting conformational adaptability while retaining overall structural integrity. Overall, the RMSD profiles indicate that the Lig-1 and Lig-2 complexes exhibited greater structural stability compared to Lig-3 and showed stability comparable to or better than the standard complex throughout the 100 ns simulation period.

The RMSF analysis was performed by molecular dynamics simulation to verify the residue-wise flexibility of the protein MPro in complex with Lig-1, Lig-2, Lig-3, and the Standard compound (Figure 7B). Most residues in all complexes fluctuated within approximately 0.5–2.5 Å, indicating overall structural stability throughout the simulation period. Among all systems, the Lig-1–MPro exhibited lower residue fluctuations, suggesting enhanced conformational stability and stable ligand accommodation within the active-site cavity. Lig-2 also showed relatively stable fluctuation patterns comparable to the Standard–MPro complex, although slightly higher fluctuations were observed compared to Lig-1. Importantly, the key active-site residues CYS145 and GLU166 exhibited lower fluctuations in both Lig-1 and Lig-2 complexes, indicating stable intermolecular interactions during the simulation. In contrast, the Lig-3 complex displayed comparatively higher fluctuations, particularly near terminal and loop regions, reflecting greater conformational flexibility. A prominent fluctuation peak reaching approximately 4–5 Å was observed near the terminal residue regions in all systems, corresponding to the naturally flexible behavior of these regions. Further, the RMSF analysis suggested that Lig-1 maintained the most stable residue fluctuation profile, while Lig-2 and Lig-3 also demonstrated prominent structural stability throughout the 100 ns simulation period.

RG analysis was performed over a 100 ns molecular dynamics simulation to evaluate the overall compactness and conformational stability of the protein in complex with Lig-1, Lig-2, Lig-3, and the standard compound. All the complexes maintained relatively stable RG trajectories throughout the simulation period, indicating preservation of the protein’s folded architecture under solvated conditions (Figure 7C). The Lig-1 complex demonstrated a highly stable compactness pattern, maintaining an average RG value around 22.2–22.4 Å, with minor fluctuations throughout the simulation. Similarly, the Lig-2 complex exhibited stable conformational stability with RG values fluctuating approximately between 22.3 and 22.5 Å, suggesting maintenance of structural compactness and stable ligand accommodation within the binding pocket. The standard complex also demonstrated consistent compactness throughout the simulation, with RG values remaining nearly between 22.4 and 22.6 Å, indicating structural stability comparable to the ligand-bound systems. In contrast, the Lig-3 complex displayed relatively higher Rg fluctuations, particularly between 40 and 80 ns, where values transiently increased up to approximately 22.7 Å. These fluctuations suggest moderate conformational flexibility and slight expansion of the protein structure during simulation; however, the system remained within an acceptable and stable compactness range.

Collectively, the RG profiles indicated that the Lig-1 and Lig-2 complexes preserved a compact and conformationally stable architecture throughout the 100 ns simulation, showing behavior comparable to the standard complex. Notably, the Lig-1 complex exhibited the least fluctuation in RG values among all systems, reflecting enhanced structural rigidity and a well-maintained protein–ligand conformation under dynamic simulation conditions.

### 2.7. Stability of Complexes Assessed with Hydrogen Bond Monitoring

**Figure 8:**
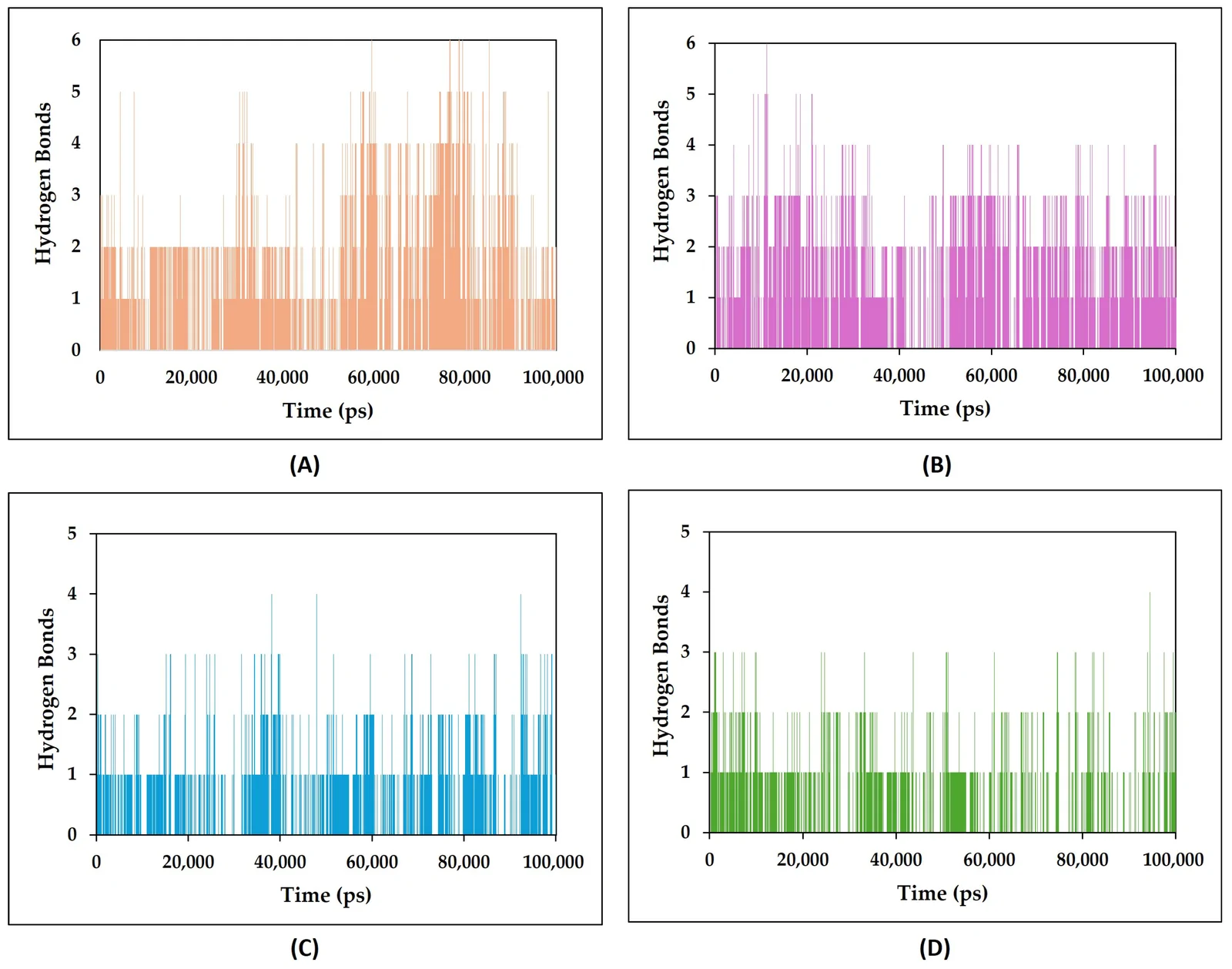
Hydrogen bond monitoring analysis of the Standard (**A**), Lig-1 (**B**), Lig-2 (**C**), and Lig-3 (**D**) complexes with MPro protein, showing the number of intermolecular hydrogen bonds maintained during the 100 ns molecular dynamics simulation.

The evaluation of hydrogen bond monitor suggests bonding between residues of protein and selected ligands. Hydrogen bond monitoring analysis was carried out during the 100 ns molecular dynamics simulation to evaluate the persistence and stability of intermolecular interactions formed between the protein and the standard ligand, Lig-1, Lig-2, and Lig-3 complexes (Figure 8A–D). The number of hydrogen bonds maintained during the simulation is an important indicator of binding stability, where a higher and more persistent hydrogen bond count generally corresponds to stronger and more stable protein–ligand interaction.

The standard complex (Figure 8A) maintained approximately two to four hydrogen bonds throughout most of the simulation period, with several intervals showing transient increases up to six hydrogen bonds. The continuous presence of hydrogen bonds with fewer dissociation events indicated stable intermolecular interactions within the binding pocket. Similarly, the Lig-1 complex (Figure 8B) demonstrated comparatively stable hydrogen bond occupancy during the entire simulation period. The complex predominantly maintained three to five hydrogen bonds, while transient increases reaching six to seven hydrogen bonds were also observed during several intervals of the trajectory. The sustained presence of multiple hydrogen bonds suggests strong and persistent intermolecular interactions, thereby contributing to enhanced stability of the Lig-1 complex. However, the Lig-2 complex (Figure 8C) showed lower hydrogen bond occupancy as compared to the Standard, generally maintaining one to three hydrogen bonds throughout the simulation. Although occasional increases in hydrogen bond number were observed, the complex showed more frequent dissociation events and reduced interaction persistence relative to the Standard and Lig-1 complexes, indicating comparatively weaker binding stability. Likewise, the Lig-3 complex (Figure 8D) maintained predominantly one to two hydrogen bonds during most of the simulation trajectory, with only occasional transient increases. Frequent fluctuations and intermittent reductions in hydrogen bond count suggested comparatively fewer stable intermolecular interactions and weaker binding persistence within the active-site region.

Overall, the hydrogen bond monitoring analysis revealed that Lig-1 and Lig-2 maintained comparatively higher and more persistent hydrogen bond occupancy throughout the 100 ns simulation period, suggesting stable intermolecular interactions and improved protein–ligand complex stability under dynamic simulated conditions.

### 2.8. Hydrogen Bonds Distance Variation Analysis for Complex Stability

**Figure 9:**
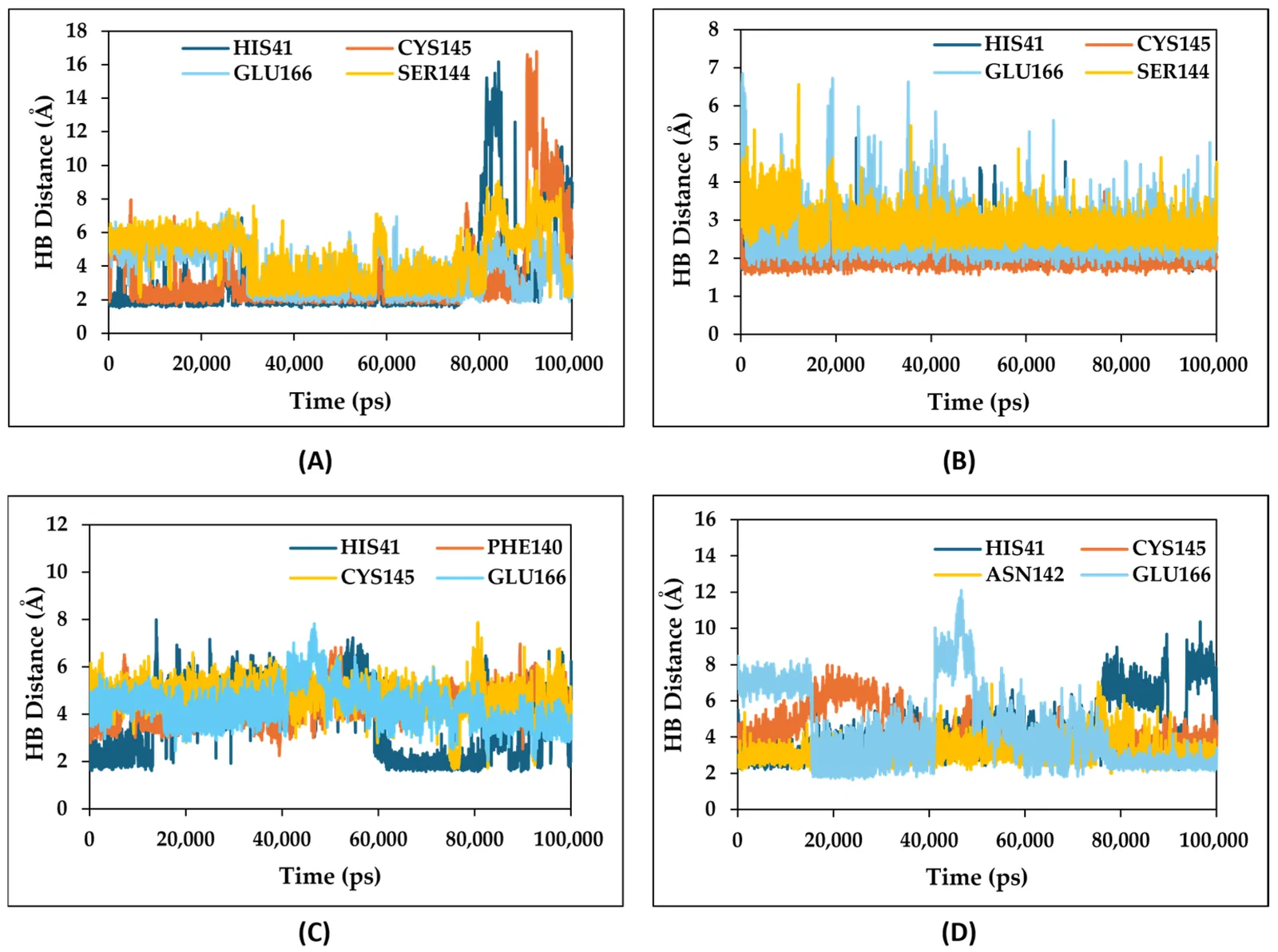
Hydrogen bond (HB) distance analysis of the Standard (**A**), Lig-1 (**B**), Lig-2 (**C**), and Lig-3 (**D**) complexes with MPro protein, showing interactions with key active-site residues during the 100 ns molecular dynamics simulation.

Variation in hydrogen bond (HB) distance analysis for amino acid residues contributing to substantial hydrogen bonds was performed throughout the 100 ns molecular dynamics simulation to investigate the strength and stability of intermolecular interactions formed between the protein with the Standard and other ligand-bound complexes. The hydrogen bond distance variations for the native Standard (Baicalein), Lig-1, Lig-2, and Lig-3-MPro complex are respectively shown in Figure 9A–D.

The standard compound formed hydrogen bond interactions mainly with HIS41, GLU166, CYS145, and SER144 residues (Figure 9A). The HIS41 and GLU166 residue average HB distances were around ~1.8–2.5 Å during most of the simulation, indicating relatively strong and stable hydrogen bonding interactions. In contrast, SER144 and CYS145 showed comparatively higher bond distances ranging approximately from 3 to 6 Å, with transient fluctuations in bond distance, which reached up to ~15–16 Å in the later stages of the simulation, suggesting comparatively weaker and unstable intermolecular interactions. However, the Lig-1–MPro complex demonstrated comparatively stable hydrogen bonding interactions throughout the simulation time (Figure 9B). To Lig-1, the MPro interacting residues HIS41, GLU166, CYS145, and SER144 maintained average HB distances of approximately 2.5–3.5 Å. Among these, CYS145 showed the shortest and most consistent bond distance around ~1.7–2.0 Å, indicating a strong and persistent interaction with Lig-1. Although GLU166 and SER144 exhibited occasional fluctuations reaching ~4–6 Å, most of the interactions remained within favorable hydrogen bonding distance ranges (~1.7–3 Å), supporting stable ligand binding. Similarly, the Lig-3–MPro complex (Figure 9D) demonstrated moderate and relatively stable hydrogen bond interactions with key active-site residues HIS41, ASN142, CYS145, and GLU166 throughout the 100 ns simulation period. The average hydrogen bond distances of HIS41 and GLU166 remained approximately within the range of 2–6 Å, indicating stable intermolecular interactions during most of the trajectory. Similarly, CYS145 and ASN142 exhibited bond distances predominantly around 4–6 Å, with moderate fluctuations and occasional transient increases. Compared to Lig-1, the Lig-3–MPro complex displayed slightly higher variations in hydrogen bond distances, suggesting comparatively reduced interaction stability within the active-site cavity. However, the majority of interactions remained within an acceptable hydrogen bonding range, supporting stable ligand accommodation throughout the simulation period. In contrast, the Lig-2 complex (Figure 9C) exhibited comparatively fewer stable hydrogen bonding interactions. The interacting residues HIS41, CYS145, PHE140, and GLU166 showed substantial fluctuations in HB distances throughout the simulation. Average bond distances varied approximately between 3 and 8 Å, while certain interactions transiently increased up to 8–12 Å, particularly for GLU166 and HIS41 during the later stages of the total simulation time. These pronounced fluctuations suggest weaker interaction persistence and comparatively reduced binding stability of the Lig-2 complex under dynamic simulation conditions. Overall, the HB distance analysis indicated that Lig-1 maintained the most stable and consistent hydrogen bonding interactions among all investigated complexes, while Lig-3 also demonstrated prominent and relatively stable intermolecular interactions, although with slightly higher fluctuations compared to Lig-1, throughout the 100 ns simulation period.

### 2.9. MM-GBSA Binding Free Energy Analysis of Ligand–Protein Complex MDS Confirmations

The binding free energy of the protein–ligand complexes was calculated to further validate the binding affinity predicted by molecular docking and molecular dynamics simulations. The MM-GBSA binding free energies obtained from the equilibrated production trajectories of two independent 100 ns molecular dynamics simulations for are presented in Table 6, providing a statistically robust estimate of the protein–ligand binding affinities. Among the investigated compounds, Lig-1–MPro exhibited the most favorable binding free energy (−47.28 ± 4.82 kcal/mol), followed by Lig-3 (−44.36 ± 4.05 kcal/mol) and Lig-2 (−42.15 ± 3.51 kcal/mol), whereas the standard ligand Baicalein–MPro complex displayed the least favorable binding energy (−38.27 ± 3.08 kcal/mol).

The lower (more negative) binding free energy values observed for Lig-1 and Lig-3, compared with the standard ligand, indicate stronger and more stable interactions with the MPro active site. Furthermore, the corresponding complex, receptor, and ligand energy components remained consistent across the analysed systems, supporting the reliability of the MM-GBSA calculations. The superior binding affinity of Lig-1 is in the agreement with its favorable docking (−CDOCKER) score and stable molecular dynamics behaviour, while Lig-3 and Lig-2 also demonstrated improved binding compared with the standard compound.

## 3. Discussion

Coronaviruses are currently classified into four genera, namely α-, β-, γ-, and δ-coronaviruses, based on genomic organization and serological characteristics [39]. SARS-CoV-2, a member of the family *Coronaviridae* and subfamily *Orthocoronavirinae*, belongs to the β-coronavirus genus. Coronaviruses, including SARS-CoV, MERS-CoV, and SARS-CoV-2, encode structurally and functionally conserved proteins involved in viral replication, among which the main protease (MPro) represents an important therapeutic target (Figure 2) [40]. SARS-CoV-2 synthesizes the polyproteins pp1a and pp1ab, which are proteolytically processed to generate 16 non-structural proteins required for viral replication and transcription. MPro mediates cleavage at 11 conserved sites within these polyproteins and is therefore essential for maturation of the viral replication machinery [41].

Structurally, MPro is a conserved dimeric cysteine protease characterized by a catalytic dyad formed by HIS41 and CYS145, residues that are critically involved in substrate recognition and catalytic activity [42,43]. Due to the absence of closely related human homologs and its indispensable role in the viral life cycle, MPro represents an attractive target for antiviral drug development. The therapeutic success of protease inhibitors against other RNA viruses, including HIV and HCV, further supports the potential of targeting MPro for SARS-CoV-2 treatment [44]. Beyond viral polyprotein processing, MPro also promotes viral survival by suppressing host antiviral immune signaling and altering host metabolic pathways, including lipid metabolism, to facilitate efficient SARS-CoV-2 replication [41,45].

Considering the multifunctional role of MPro in both viral replication and immune evasion, inhibition of this protease could simultaneously suppress viral maturation and interfere with mechanisms that support viral persistence within the host. In the present study, molecular docking and molecular dynamics simulations were employed to evaluate the stability and inhibitory potential of the selected ligands against the MPro active site. The investigated complexes demonstrated stable binding interactions with key catalytic residues, particularly HIS41, CYS145, and GLU166, which are known to play essential roles in ligand recognition and enzymatic activity.

The generated pharmacophore models highlight the importance of hydrogen bond acceptors, hydrophobic aromatic regions, and aromatic ring interactions for stable ligand binding within the SARS-CoV-2 MPro active-site cavity. Among the generated hypotheses, the five-feature pharmacophore model AAHaromHaromR demonstrated the best predictive performance with favorable AUC, sensitivity, and specificity values, indicating reliable discrimination between active and inactive compounds. The validated model was subsequently utilized for virtual screening using the ZINCPharmer database, resulting in 483 initial hit compounds [46]. Following duplicate removal, energy minimization, and fix bad valencies filtering, 230 compounds exhibiting essential pharmacophoric features were selected for further pharmacophore mapping internally through Biovia Discover studio. Based on the internal pharmacophore mapping results screened, 49 compounds showed a fit value range from 3.5 to 0. To filter out further compounds with fit values ranging from 3.5 to 1 were considered to exhibit acceptable geometric and chemical complementarity with the validated model [47].

This filtering strategy ensured the prioritization of 21 compounds with optimal spatial and chemical feature alignment, thereby increasing the likelihood of identifying biologically relevant inhibitors. These findings suggest that the selected pharmacophore model provides a reliable framework for identifying potential computational lead compounds with promising binding characteristics [48,49].

The sequential application of drug-likeness filters (Lipinski and Veber rules), PAINS screening, and ADMET evaluation effectively enriched the screening library by eliminating compounds with unfavorable physicochemical or pharmacokinetic properties. Consequently, the 15 compounds exhibiting favorable developability profiles were prioritized for subsequent molecular docking and molecular dynamics simulations, thereby increasing the likelihood of identifying biologically relevant and drug-like lead candidates. The molecular docking (−CDOCKER Score and MM-GBSA) of the 15 selected ligands revealed that Lig-1 followed by Lig-2 exhibited stronger binding affinities and more favorable interaction profiles within the SARS-CoV-2 MPro active-site cavity compared to the other investigated compounds (Table 4 and Table S1). All three ligands established stable interactions with crucial catalytic residues, including HIS41, CYS145, GLU166, MET49, and MET165, which are essential for the proteolytic activity of MPro. Among the investigated compounds, Lig-1 demonstrated the most stable binding orientation and interaction persistence, suggesting enhanced inhibitory potential against MPro activity. To further validate the docking results, the shortlisted compounds were independently evaluated using the AutoDock Vina docking algorithm. The obtained binding affinities were consistent with the −CDOCKER score and MM-GBSA rankings, with the lead compounds exhibiting stronger predicted binding than the reference ligand Baicalein. The agreement among these independent computational approaches enhances the confidence in the identified lead compounds and supports the robustness of the docking methodology employed in this study. To place the identified inhibitors in the context of experimentally validated SARS-CoV-2 MPro inhibitors nirmatrellvir, Ensitrelvir (S-217622), GC376, Boceprevir and Baicalein, a comparative summary of representative inhibitors, including their reported key interacting residues, is provided in Table S4. The observed interaction patterns of Lig-1, Lig-2 and Lig-3 share several conserved active-site residues with established MPro inhibitors, supporting the predicted binding mode and potential inhibitory activity of the identified compounds. The interaction pattern observed for Lig-1, Lig-2 and Lig-3 overlaps with the binding mode of several experimentally validated SARS-CoV-2 MPro inhibitors, particularly through interactions with conserved catalytic and substrate-binding residues such as His41, Cys145, Glu166, Met165, and Gln189. The conservation of these interactions suggests that the identified compounds may exploit a common molecular recognition mechanism within the MPro active site, supporting their predicted binding mode. Therefore, the virtual screening through docking, ADMET and pharmacokinetic drug likeness prediction-integrated funnel suppresses false positives, improves and prioritizes only those ligands that can maintain affinity with MPro, and increases the chance that the final hits will translate into stable, safe chemical matter suitable for further experimental downstream validation and lead optimization [50,51].

The stable interactions of ligands with catalytic pocket residues were further supported by molecular dynamics simulation analyses, indicating stable complex formation throughout the 100 ns simulation period. The interaction of ligands with binding pocket residues including catalytic dyad residues would impact cleavage of viral polyproteins. Owing to its determining role in the proteolysis of viral polyproteins, MPro of the FIPV has been established as the preferred target combating its virulence [40].

The MPro catalytic dyad is pivotal for the enzymatic activity of MPro, facilitating the cleavage of viral polyproteins into functional proteins essential for viral replication and assembly. Upon takeover of a host’s transcriptional machinery, the host cell expresses two overlapping polyproteins, pp1a and pp1ab, which cleave through coronavirus-encoded proteases—papain-like protease (PLpro) and MPro. The cleavage of pp1a and pp1ab forms 16 nonstructural proteins that play roles in viral replication [38,52,53].

During molecular dynamics simulation, the RMSD, RMSF, and RG analyses collectively demonstrated that Lig-1 and Lig-2 maintained stable conformations and compactness throughout the 100 ns simulation period. Notably, Lig-1 exhibited lower structural fluctuations, reduced residue flexibility, and more persistent hydrogen bonding interactions compared to the other investigated complexes, indicating enhanced conformational stability within the active-site cavity.

Ideally, RMSD values would be zero; however, due to statistical uncertainties, it is not possible for a protein to have an RMSD of zero [54]. The RMSF quantifies the overall structural deviations over time and measures the root mean square fluctuations of individual amino acid residues, providing insight into the dynamic behavior and flexibility of these residues during simulations [55]. The magnitude of RG inversely correlates with protein stability, where a larger RG indicates a less stable, more expanded structure. Therefore, the RMSF, RMSD and RG profiles of all investigated complexes were comparable to those of the reference standard, Lig-1–MPro and Lig-2-MPro complexes followed by Lig-3, exhibiting relatively lower residue fluctuations and indicating favorable structural stability throughout the simulation period [56].

Furthermore, hydrogen bond monitoring and hydrogen bond distance analyses revealed that Lig-1 consistently maintained stable intermolecular interactions with catalytic pocket residues, suggesting stronger binding persistence under dynamic simulated conditions. However, Lig-3 also exhibited considerable interaction stability; however, slightly higher fluctuations and lower hydrogen bond occupancy were observed compared to Lig-1. Conversely, Lig-2 showed comparatively weaker interaction retention and increased conformational flexibility during the simulation period. Greater variation in hydrogen bond distances reflects reduced stability of ligand interactions within the active-site pocket, whereas hydrogen bond distances close to or below 2.5 Å are typically associated with stronger and more stable protein–ligand binding interactions [57,58]. Overall, the post MDS MM-GBSA analysis corroborated the molecular docking and MDS results, confirming that the identified lead compounds possess predicted better binding affinity and complex stability relative to the standard compound. Based on the integrated computational analyses, Lig-1 emerged as the most promising candidate, followed by Lig-3 and Lig-2, warranting further experimental validation as potential inhibitors of the target SARS-CoV-2 Mpro protein.

This study is subject to several limitations. The identified lead compounds were prioritized using computational approaches, and therefore require experimental biochemical and antiviral validation to confirm their inhibitory activity. The analysis was performed exclusively on SARS-CoV-2 MPro using a single crystallographic structure (PDB ID: 6M2N), and cross-coronavirus inhibitory potential as well as protein conformational diversity were not investigated. However, two independent 100 ns molecular dynamics simulations were conducted to improve reliability; longer simulations and enhanced sampling techniques may provide additional insights into ligand stability and binding dynamics. Furthermore, while Lipinski, Veber, PAINS, and ADMET filters were applied to prioritize drug-like compounds, additional developability assessments such as synthetic accessibility, aggregation propensity, and reactive group analysis were beyond the scope of this study. Finally, although the pharmacophore model demonstrated good predictive performance, validation was performed using a curated dataset of 77 compounds, and evaluation on larger and more diverse datasets could further establish its generalizability. Therefore, the identified compounds should be considered promising computational lead candidates requiring further experimental validation. Although the catalytic residues of MPro are conserved among representative alpha- and betacoronaviruses, conservation of sequence does not necessarily imply conservation of ligand recognition because variations in the surrounding binding-pocket architecture may influence ligand binding. Therefore, the present computational analyses should not be interpreted as demonstrating broad-spectrum antiviral activity. Instead, the identified compounds should be regarded as computational lead candidates targeting SARS-CoV-2 MPro that warrant further computational, biochemical, and antiviral evaluation against additional coronavirus MPro proteins.

## 4. Materials and Methods

BIOVIA Discovery Studio Client (v24.1.0.321712, Dassault Systèmes, San Diego, CA, USA) was used for all computational investigations. Molecular dynamics simulations were performed using the simulation protocols available within the BIOVIA Discovery Studio platform.

### 4.1. Multiple Sequence Alignment

The protein sequence of MPro from ten coronaviruses were retrieved from NCBI with the accession numbers HCoV-NL63 Accession No. XPR31551, HCoV-229E Accession No. WDE18043, FCoV Accession No. QSL97047, PEDV Accession No. WJJ67095, SARS-CoV-2 Accession No. 5R7Z_A, SARS-CoV Accession No. AFR58685, HCoV-HKU1 Accession No. WDE18948, HCoV-OC43 Accession No. YP_009555238, BCoV Accession No. UZT75375, and MERS Accession No. AGV08377. Multiple sequence alignment was performed using Geneious Prime V.9.0 [59].

### 4.2. Preparation of Protein, Energy Optimization and Sphere Generation

The X-ray crystallographic structure of SARS-CoV-2 main protease (MPro) (PDB ID: 6M2N) was retrieved from the RCSB Protein Data Bank (https://www.rcsb.org/structure/6M2N, accessed on 10 October 2025). The selected structure corresponds to the monomeric biological unit of SARS-CoV-2 MPro with a sequence length of 306 amino acids and a resolution of 2.20 Å. The crystal structure contains the co-crystallized non-covalent inhibitor Baicalein (5,6,7-trihydroxy-2-phenyl-4H-chromen-4-one), which was used for binding-site definition and docking protocol validation. Protein preparation was performed using the Prepare Protein protocol implemented in BIOVIA Discovery Studio Client with the CHARMm force field.

During preparation, unnecessary heteroatoms, crystallographic artifacts, and the co-crystallized ligand were removed after binding-site definition, while water molecules located beyond 5 Å from the binding site were deleted. Hydrogen atoms were added according to physiological protonation states, missing atoms and side chains were repaired, unresolved residues were corrected where applicable, and the protein structure was subsequently subjected to energy minimization to relieve steric clashes and optimize the geometry for molecular docking [60].

Following protein preparation, the Define and Edit Binding Site protocol was employed to generate the receptor-binding sphere using the centroid of the co-crystallized Baicalein molecule. The binding site was defined using the coordinates (−33.2182, −65.3489, 41.2083) with a radius of 6.854 Å. The co-crystallized ligand was subsequently removed while retaining the defined binding sphere to enable docking of the screened compounds within the experimentally validated active site [40]. To validate the docking protocol, the co-crystallized ligand was re-docked into the prepared receptor using identical docking parameters. The docking protocol was considered successfully validated when the root mean square deviation (RMSD) between the crystallographic pose and the re-docked pose was ≤2.0 Å, confirming the reliability of the docking procedure for reproducing the experimentally observed binding mode.

### 4.3. Ligand Preparation

The screened compounds obtained after pharmacophore-based virtual screening were prepared using the Prepare Ligands protocol in BIOVIA Discovery Studio v24.1.0.321712. During ligand preparation, bond orders and valencies were corrected, appropriate protonation states were assigned, hydrogen atoms were added, and CHARMm-compatible atom types and partial charges were automatically assigned. The ligands were subsequently subjected to geometry optimization and energy minimization prior to molecular docking, ensuring chemically valid and energetically favorable conformations for subsequent docking and molecular dynamics simulations.

### 4.4. Pharmacophore Model Development and Validation

The interaction-based pharmacophore model was generated using BIOVIA Discovery Studio v24.1.0.321712. The protocol was set to generate selective pharmacophore hypotheses by directly utilizing the non-bonded interactions between a receptor ligand complex, thereby identifying the essential chemical and geometric features responsible for molecular recognition. The generated pharmacophore features include hydrogen bond acceptors and hydrogen bond donors, hydrophobic and aromatic interactions, positive and negative ionizable features, halogen interactions, sulfur interactions, amide groups, and other interactions available within the BIOVIA framework. Multiple candidate pharmacophore models were subsequently generated from these interaction-derived features and subsequently ranked according to their predicted selectivity; they were retained using a Genetic Function Approximation (GFA) model of Biovia Discovery Studio [61].

The experimentally determined SARS-CoV-2 MPro–co-crystallized ligand complex was employed as the structural template for pharmacophore generation [38].

The ligand binding site was defined according to the position of the co-crystallized ligand and referenced to the coordinates (−33.2182, −65.3489, 41.2083), with a sphere radius of 6.854 Å.

The Interaction pharmacophore generation method was set as follows: a maximum of 10 pharmacophores with minimum and maximum 4 and 6 features, respectively. The minimum interfeature distance was specified as 2 Å, while steric constraints were represented as Excluded Volumes to account for 3D architecture of the receptor binding pocket. Pharmacophore generation considered the selected non-bond interaction categories, including conventional hydrogen bonds, π–cation interactions, π–anion interactions, π–donor interactions, π–π stacking interactions, salt bridges, attractive charge interactions, and other interaction types available within the protocol.

To evaluate the predictive performance of the generated pharmacophore models, BIOVIA Discovery Studio pharmacophore validation was employed using a validation dataset comprising 38 experimentally active and 39 experimentally inactive compounds retrieved from the ChEMBL database (total 77 compounds). The active and inactive compounds were curated by filtering molecules reported against SARS-CoV-2 MPro with experimentally determined pIC_50_ values. Compounds with pIC_50_ ≥ 5.5 were classified as active, whereas compounds with pIC_50_ ≤ 4.0 were classified as inactive, while compounds with intermediate activities were excluded from the validation set to improve the discriminatory power of the pharmacophore model.

The protocol automatically performed receiver operating characteristic (ROC) analysis together with the corresponding true positives (TP), false positives (FP), true negatives (TN), false negatives (FN), sensitivity, specificity, and area under the ROC curve (AUC) values. Sensitivity (true positive rate) was calculated as TP/(TP + FN), whereas specificity (true negative rate) was calculated as TN/(TN + FP). The false positive rate was calculated as FP/(FP + TN). The AUC value was used as a threshold independent measure of model discrimination ability, with higher AUC values indicating superior predictive performance. Pharmacophore models having high AUC values together with a balanced sensitivity and specificity were considered the most reliable for distinguishing active from inactive and were chosen for subsequent virtual screening.

The validated pharmacophore model was subsequently employed for pharmacophore-based virtual screening using the external ZINCPharmer web server (https://zincpharmer.csb.pitt.edu/, accessed on 13 December 2025). ZINCPharmer utilizes the Pharmer search engine to rapidly screen approximately 230 million molecular conformations from the ZINC purchasable compound database against a defined pharmacophore model feature. To enrich the screening library with compounds having drug likeness properties, a molecular weight filter of 350–500 Da was applied [62]. The resulting ligands from ZINCPharmer were then subjected to the Prepare ligand method to remove duplicate ligands, energy minimization, and to fix bad valencies among filtered ligands. Further, after preparation of ligands, filtered compounds were then internally re-evaluated using the Ligand Pharmacophore Mapping protocol (internal mapping) from BIOVIA Discovery Studio to verify their ability to reproduce the validated pharmacophore features within the software environment [62].

This internal (Ligand Pharmacophore Mapping) mapping step served as a secondary validation of the external mapping (ZINCPharmer) results and enabled the selection of compounds exhibiting the highest pharmacophore fit values. Following this refinement process, unique compounds were employed for Lipinski, ADMET and PAINS analyses.

### 4.5. Drug-Likeness and ADMET Evaluation with PAINS

The compounds shortlisted through pharmacophore-based virtual screening were further evaluated for their drug-likeness and pharmacokinetic properties prior to molecular docking using the ADMET prediction protocol implemented in BIOVIA Discovery Studio v24.1.0.321712. The evaluated parameters included human intestinal absorption, aqueous solubility, blood–brain barrier penetration, cytochrome P450 (CYP2D6) inhibition, and hepatotoxicity, enabling the early identification of compounds with favorable pharmacokinetic profiles. In parallel, the compounds were assessed for compliance with established drug-likeness criteria, including Lipinski’s Rule of Five and Veber’s rule filters. Lipinski’s rule evaluates molecular weight (≤500 Da), LogP (≤5), hydrogen bond acceptors (≤10), and hydrogen bond donors (≤5), whereas Veber’s rule considers rotatable bonds (≤10) and molecular polar surface area (MPSA ≤ 140 Å^2^) as indicators of oral bioavailability to identify compounds with drug-like physicochemical characteristics. The compounds exhibiting acceptable ADMET properties and satisfying the predefined drug-likeness criteria were retained for subsequent molecular docking and molecular dynamics analyses.

### 4.6. Molecular Docking and Binding Energy Calculation of Selected Ligands

To evaluate the binding affinity and interaction profiles of the drug-like compounds selected after drug-likeness and ADMET screening, molecular docking was performed using the −CDOCKER protocol implemented in BIOVIA Discovery Studio v24.1.0.321712. −CDOCKER is a CHARMm-based molecular docking algorithm that employs a simulated annealing molecular dynamics approach while allowing full ligand flexibility through variations in torsion angles, bond angles, and bond lengths to generate energetically favorable binding conformations. For each ligand, the −CDOCKER protocol was configured to generate 50 docking poses. These conformations were evaluated and ranked according to their −CDOCKER interaction energies, and the top 10 docking poses were retained. Among these top 10 poses for each docked ligand, the docking pose exhibiting the highest −CDOCKER score was subsequently selected for protein–ligand interaction analysis and molecular dynamics simulations… Additionally, AutoDock Vina docking based on the Lamarckian genetic algorithm was performed independently to validate binding orientations and binding affinities. AutoDock Vina predicted binding free energies and was used to cross-validate the ligand ranking score to ensure robustness and consistency of docking outcomes across multiple scoring functions [63,64].

Protein–ligand interactions were visualized using Discovery Studio to identify key hydrogen bonds, hydrophobic interactions, π–π stacking, van der Waals contacts, and other non-covalent interactions within the MPro active site. To further estimate the binding affinity of the docked complexes prior to molecular dynamics simulations, MM-GBSA binding energies were calculated using the Calculate Binding Energies protocol implemented in BIOVIA Discovery Studio [24,25,40,62]. The MM-GBSA binding energies reported in this study correspond to the energy estimates generated by the BIOVIA Discovery Studio Calculate Binding Energies method used for the relative ranking and comparison of protein–ligand complexes within the same computational workflow rather than as absolute experimental binding free energies.

### 4.7. Molecular Dynamic Simulation (MDS) Analysis

MDS were carried out using BIOVIA Discovery Studio v24.1.0.321712. Based on docking score, binding energy, and conformational pose analysis, the best complexes (MPro–ligand) were selected for MDS. The best predicted top hits of ligands with respect to MPro were selected to perform 100-nanosecond (ns) analyses through the standard dynamics cascade method. To generate the molecular topology files for the MPro and MPro–ligand complex and to create the topology of ligands, the CHARMm force field was used. The solvation system consists of an explicit boundary TIP 3-point water solvent model, an orthorhombic box with a minimal distance of 7 Å between the protein surface and the edge of the box neutralized with the inclusion of cation-type sodium (Na) and anion-type chloride (Cl) counter ions [65].

After solvation of the protein and complex, the standard dynamics cascade method was employed. For energy minimization, the steepest descent (minimization 1) was used for 1000 steps with RMS gradient 1 and conjugate gradient (minimization 2) for 4000 steps with RMS gradient 0.1. Both minimization algorithms were used for a total of 5000 steps. The heating phase was performed using a simulation time of 100 picoseconds (ps) with a time step of 2 fs; for immersion, the initial temperature was 50 and the target temperature was 300 K, with a save results interval of 10 ps. The reference temperature was 300 K, and the reference pressure was 1.0 bar for the NPT (isothermal–isobaric) ensemble. The equilibration phase was carried out for a 2000 ps simulation run with a 2 fs (femtosecond) time step, and the save result interval was 10 ps to generate the restart file for further equilibration of the complex. The Particle_Mesh_Ewald (PME) algorithm was used for long-range electrostatic interactions with fourth-order cubic interpolation and a kappa 0.34 Å grid spacing. The advanced dynamic integrator used the Leapfrog Verlet algorithm with the applied shake constraint. The harmonic restraint with force constant 10 was applied to the backbone of protein–ligand complexes during heating and equilibration phases. The explicit solvent model was used with a dielectric constant of 1, a non-bond list radius cutoff of 14 Å, in which the non-bond higher cutoff distance is 12 A and the non-bond lower cutoff distance is 10 Å. The production step of a standard dynamic cascade of MDS was carried out for 100,000 ps (100 nanosecond (ns)) with a save result interval step for 20 ps, which makes a total of 5000 frames (confirmations). For each protein–ligand complex, two independent molecular dynamics simulations were performed for 100 ns.

Several key parameters were analyzed to understand the dynamics of the system. To analyze trajectory, root mean square deviation (RMSD), root means square fluctuation (RMSF), and radius of gyration (RG) were calculated to measure the overall stability of the protein in the absence and presence of ligand by tracking how much the atoms moved relative to a reference structure [66,67]. The stability of the complex in each frame was further confirmed by monitoring established hydrogen bonds between protein and ligand interacting atoms. The stability of the complex is indicated by the highest potential inhibitor from the stable protein–ligand complex through BIOVIA Discovery Studio v24.1.0.321712 [40].

### 4.8. Post-MDS MM-GBSA Binding Free Energy Calculation

Binding free energy calculations were performed using the Calculate Binding Energies for a Trajectory protocol implemented in BIOVIA Discovery Studio v24.1.0.321712. Prior to energy calculations, the stability of each protein–ligand complex was confirmed through analysis of the MDS trajectories (RMSD, RMSF, Rg, and hydrogen bond profiles), ensuring that the ligands remained stably bound within the active site throughout the production simulation. Two independent 100 ns MDS were performed for each complex, and MM-GBSA calculations were conducted separately for each trajectory using snapshots extracted from the equilibrated production phase. Generally, the binding free energy (ΔG*_Bind_*) between a protein and a ligand in solvent is calculated as follows:

ΔG*_Bind_* = G*_Complex_* − (G*_Protein_* + G*_Ligand_*)

where G*_Complex_* denotes the total free energy of the protein–ligand complex, and G*_Protein_* and G*_Ligand_* represent the total free energies of the isolated protein and ligand in the solvent, respectively. The reported binding free energies represent the mean ± standard deviation (SD) obtained from the independent simulations. No trajectories were excluded from the analysis, thereby minimizing potential bias and providing a robust estimation of ligand binding affinity.

## 5. Conclusions

The present computational investigation identified Lig-1, followed by Lig-3, as promising computational lead compounds targeting the SARS-CoV-2 MPro, based on their favorable predicted binding affinity, stable interactions with key catalytic residues, and sustained structural stability throughout the 100 ns molecular dynamics simulations. The integrated pharmacophore modeling, molecular docking, molecular dynamics simulations, and MM-GBSA binding free energy analyses consistently supported the stable binding of these compounds within the active-site cavity of SARS-CoV-2 MPro. These findings provide a strong computational foundation for the rational design and optimization of MPro-targeted antiviral agents. However, because the present study was conducted using only the SARS-CoV-2 MPro structure, the activity of these compounds against MPro enzymes from other coronaviruses cannot be inferred from the current data. Therefore, comprehensive in vitro and in vivo studies, together with evaluation against MPro proteins from additional coronaviruses, are necessary to validate their biological activity, antiviral efficacy, and therapeutic potential.

## Figures and Tables

**Table 1:** Validation statistics of the generated pharmacophore models.

Pharmacophore Model Validation with Known Actives/Inactives									
Pharmacophore Model (PPM)	Total Actives	Total Inactives	True Positives	True Negatives	False Positives	False Negatives	Sensitivity	Specificity	AUC ROC
PPM-1	38	39	31	24	15	7	0.81579	0.61538	0.781
PPM-2	38	39	30	21	18	8	0.78947	0.53846	0.738
PPM-3	38	39	37	5	34	1	0.97368	0.12821	0.513
PPM-4	38	39	37	6	33	1	0.97368	0.15385	0.562
PPM-5	38	39	37	7	32	1	0.97368	0.17949	0.521
PPM-6	38	39	36	8	31	2	0.94737	0.20513	0.678
PPM-7	38	39	37	7	32	1	0.97368	0.17949	0.529
PPM-8	38	39	32	19	20	6	0.84211	0.48718	0.686
PPM-9	38	39	34	9	30	4	0.89474	0.23077	0.508
PPM-10	38	39	30	19	20	8	0.78947	0.48718	0.700

**Table 2:** Drug-Likeness Characteristics Evaluated Using Lipinski’s Rule of Five, Veber’s Rule, and PAINS Filter.

S. No	Compound ID	HA	HD	M.Wt.	ALogP	Rotatable Bonds	MPSA (Å^2^)	PAINS
**1**	ZINC95477953	9	2	475.493	5.586	5	112	0
**2**	ZINC79497869	9	4	432.429	3.797	5	136.63	0
**3**	ZINC95480156	10	4	488.492	4.456	6	145.86	0
**4**	ZINC95476071	8	2	429.425	5.403	2	102.77	0
**5**	ZINC95478185	10	4	488.492	4.456	5	145.86	0
**6**	ZINC95473654	8	3	461.467	5.36	4	123	0
**7**	ZINC95473725	8	4	417.414	5.151	2	124.77	0
**8**	ZINC95475599	9	2	473.477	5.405	3	112	0
**9**	ZINC95475957	8	3	431.441	5.377	3	113.77	0
**10**	ZINC08792368	8	1	507.537	4.859	7	91.68	0
**11**	ZINC60353723	7	0	418.491	3.65	4	92.26	0
**12**	ZINC13135380	6	1	433.496	4.946	10	69.93	0
**13**	ZINC06409441	9	1	500.59	2.869	7	147.34	0
**14**	ZINC72066827	7	0	405.45	3.454	4	100.47	0
**15**	ZINC25540664	10	1	489.432	−3.344	4	197.99	0

HA: hydrogen acceptor; HD: hydrogen donor; MPSA: Molecular polar surface area; M.Wt.: Molecular weight.

**Table 3:** ADMET Prediction Profiles of the selected fifteen drug-like compounds.

S. No	Compound ID	Solubility Level	BBB Level	CYP2D6 Prediction	Hepatotoxicity Prediction	PPB Prediction	Absorption Level
**1**	ZINC95477953	1	4	FALSE	TRUE	TRUE	2
**2**	ZINC79497869	1	4	FALSE	TRUE	FALSE	2
**3**	ZINC95480156	1	4	FALSE	TRUE	FALSE	2
**4**	ZINC95476071	1	4	FALSE	TRUE	TRUE	1
**5**	ZINC95478185	1	4	FALSE	FALSE	FALSE	2
**6**	ZINC95473654	1	4	FALSE	FALSE	TRUE	2
**7**	ZINC95473725	1	4	FALSE	TRUE	FALSE	2
**8**	ZINC95475599	1	4	FALSE	TRUE	TRUE	2
**9**	ZINC95475957	1	4	FALSE	TRUE	TRUE	2
**10**	ZINC08792368	1	4	FALSE	FALSE	TRUE	1
**11**	ZINC60353723	1	1	FALSE	TRUE	TRUE	0
**12**	ZINC13135380	2	1	FALSE	FALSE	TRUE	0
**13**	ZINC06409441	2	4	FALSE	TRUE	TRUE	0
**14**	ZINC72066827	1	2	FALSE	FALSE	TRUE	0
**15**	ZINC25540664	3	4	FALSE	TRUE	TRUE	3

Solubility level: 0 = extremely low, 1 = very low but possible, 2 = low, 3 = good. BBB level: 1 = high penetration, 2 = medium penetration, and 4 = undefined/very low penetration. CYP2D6 prediction: FALSE = non-inhibitor. Hepatotoxicity prediction: FALSE = non-hepatotoxic, TRUE = hepatotoxic. PPB prediction: TRUE = plasma protein binding ≥ 90%, FALSE = plasma protein binding < 90%. Absorption level: 0 = good, 1 = moderate, 2 = low, and 3 = very low human intestinal absorption.

**Table 4:** Top residues of the binding pocket include catalytic dyad residues CYS145 and HIS41, exhibiting favorable interactions with the 15 selected ligands.

Top 5 Residues with Favorable Interactions (5)							
Residue	Favorable	Unfavorable	Hydrogen Bond	Charge	Hydrophobic	Halogen	Other
A:MET49	184	1	23	0	177	0	26
A:CYS145	182	0	28	0	154	0	55
A:HIS41	167	0	53	5	150	0	8
A:MET165	161	0	18	0	151	0	19
A:GLU166	145	1	143	2	0	0	2

**Table 5:** Comparative docking scores, pharmacophore fit values, RMSD, and MM-GBSA binding free energies of the top three ZINC compounds out of 21 selected compounds.

S. No.	Compound ID	Structure	Fit Value	RMSD (Å)	CDOCKER Score	Pre MDS-MM-GBSA (Kcal/mol)	AutoDoc Vina Binding Affinity (Kcal/mol)
**1**	ZINC95473654	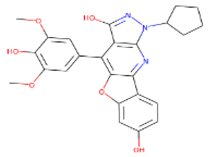	2.69159	1.25	54.60	−121.86	−8.7
**2**	ZINC95473725	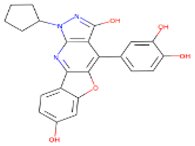	2.63669	1.52	50.83	−102.47	−8.6
**3**	ZINC08792368	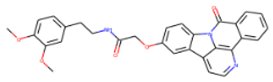	1.96643	1.43	52.73	−97.73	−8.2
**4**	Standard (Baicalein)	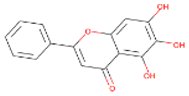	**-**	0.68	34.23	−90.02	−7.5

**Table 6:** The average binding free energies and energy components (MM-GBSA) obtained from two independent 100 ns molecular dynamics simulations.

Complex	Binding Energy ΔG*_Bind_* (kcal/mol)	Complex Energy G*_Complex_* (kcal/mol)	Receptor Energy G*_Protein_* (kcal/mol)	Ligand Energy G*_Ligand_* (kcal/mol)
Lig-1–MPro	−47.28 ± 4.82	−9989.74 ± 11.93	−10,039.91 ± 13.84	102.89 ± 0.68
Lig-2–MPro	−42.15 ± 3.51	−9969.84 ± 11.24	−10,012.88 ± 13.41	100.89 ± 0.34
Lig-3–MPro	−44.36 ± 4.05	−9978.65 ± 10.76	−10,024.53 ± 14.92	101.52 ± 0.57
Standard–MPro	−38.27 ± 3.08	−9954.22 ± 10.97	−9993.86 ± 12.18	101.37 ± 1.22

## Data Availability

The raw data supporting the conclusions of this article will be made available by the authors upon request.

## Supplementary Materials

The following supporting information can be downloaded at: https://www.mdpi.com/article/10.3390/ijms27177684/s1, https://doi.org/10.6084/m9.figshare.29298449.v1. References [68,69,70,71] are cited in the Supplementary Materials.
