# Supplementary material for: Identification of Potential SARS-CoV-2 Main Protease (MPro) Inhibitors Through Pharmacophore Modeling, Molecular Docking, and Molecular Dynamics Simulation Approaches
Source: Int J Mol Sci. 2026 Aug 27;27(17):7684. doi: 10.3390/ijms27177684 (PMC13566167; doi:10.3390/ijms27177684)
Supplement: Supplementary file 1 [file ijms-27-07684-s001.zip › ijms-4461880-supplementary.pdf]

Supporting Information/Supplementary Material

Identification of Potential SARS-CoV-2 Main Protease (Mpro) Inhibitors through Pharmacophore Modeling, Molecular Docking, and Molecular Dynamics Simulation Approaches

Mohd Yasir Khan [1], Farah Maarfi [1], Abid Ullah Shah [2], Nithyadevi Duraisamy [1], Mohammed Cherkaoui [1] and Maged Gomaa Hemida [2,*]

[1] Department of Digital Engineering and Artificial Intelligence, College of Science, Long Island University, Brooklyn, NY 11201, USA; mohd.yasirkhan@liu.edu (M.Y.K); farah.maarfi@liu.edu (F.M.); nithyadevi.duraisamy@liu.edu (N.D.); mohammed.cherkaoui@liu.edu (M.C.)

[2] Department of Veterinary Biomedical Sciences, College of Veterinary Medicine, Long Island University, 720 Northern Boulevard, Brookville, NY 11548, USA; abidullah.shah@liu.edu (A.U.S)

* Correspondence: maged.hemida@liu.edu (M.G.H); Tel.: +1-516-299-3650

Table S1. Pharmacophore-based selection of 21 ZINC compounds (from 49) with fit values between 3.5 and 1.0 for Mpro docking. The table lists compound IDs, structures, fit values, and RMSD (Å).

Table S2. Canonical SMILES of the Fifteen Selected Drug-Like Compounds.

Table S3. Best Docking Results of the 15 Shortlisted Compounds Based on MM-GBSA Binding Energy, −CDOCKER Score, and AutoDock Vina Binding Affinity.

Table S4: Comparison of Experimentally Validated SARS-CoV-2 MPro Inhibitors and the Computationally Identified Lead Compounds (Lig-1, Lig-2 and Lig-3), Highlighting Key Binding Residues and Experimental Status.

Figure S1. Receiver operating characteristic (ROC) curves of ten selected ligands (Ligand_01–Ligand_10) showing their classification performance. The x-axis represents the false positive rate (1 − specificity) and the y-axis represents the true positive rate (sensitivity). The area under the curve (AUC) and cutoff values are indicated for each ligand, reflecting their predictive accuracy. Among them, Ligand_01 demonstrated comparatively better ROC performance and AUC and was therefore selected for further evaluation.

Figure S2. Two-dimensional (2D) protein–ligand interaction diagrams of the standard and selected ZINC compounds within the active site of the target protein. (A) Standard (Reference) ligand Baicalein, (B) ZINC95473654, (C) ZINC95473725, and (D) ZINC08792368. The diagrams illustrate key interactions including conventional hydrogen bonds, carbon hydrogen bonds, π–π stacking, π–alkyl, π–sulfur, and van der Waals interactions with surrounding amino acid residues. These interactions contribute to the stability and binding affinity of the ligands within the binding pocket of Mpro.

Table S1. Pharmacophore-based selection of 21 ZINC compounds (from 49) with fit values between 3.5 and 1.0 for Mpro docking. The table lists compound IDs, structures, fit values, and RMSD (Å).

S. No.	Compound ID	Structure	Fit Value	RMSD (Å)
1	ZINC95477953	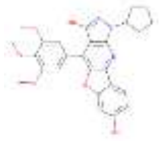	3.51045	1.43
2	ZINC79497869	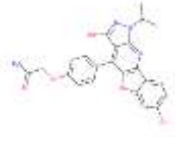	3.48168	1.47
3	ZINC95480156	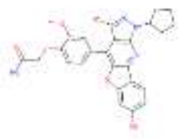	3.47317	1.43
4	ZINC95476071	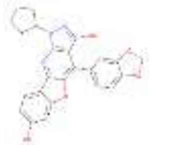	3.45944	1.47
5	ZINC79497740	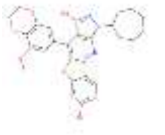	3.45922	1.38
6	ZINC95478185	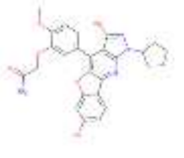	3.3623	1.43
7	ZINC95472987	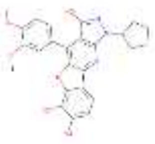	3.23349	1.37
8	ZINC95472700	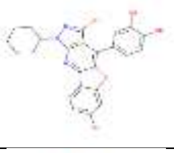	2.70913	1.51
9	ZINC95473654	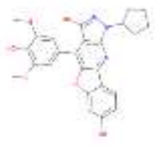	2.69159	1.43

10	ZINC95473725	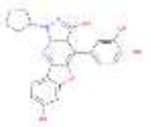	2.63669	1.52
11	ZINC95475599	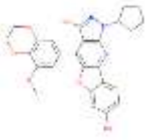	2.51441	1.34
12	ZINC95475957	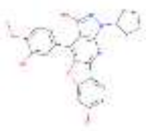	2.45731	1.43
13	ZINC95480636	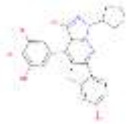	2.43252	1.43
14	ZINC79497947	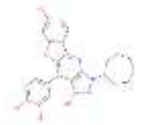	2.40423	1.43
15	ZINC08792368	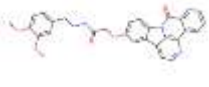	1.96643	1.43
16	ZINC60353723	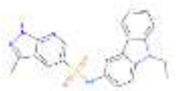	1.90943	1.56
17	ZINC13135380	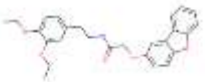	1.70556	1.13
18	ZINC06409441	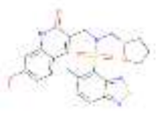	1.5154	1.27
19	ZINC72066827	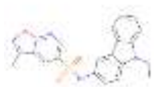	1.27094	1.56
20	ZINC25540664	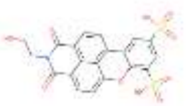	1.20868	1.31
21	ZINC59459221	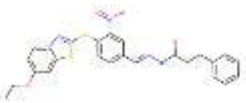	1.01992	1.29

Table S2. Canonical SMILES of the Fifteen Selected Drug-Like Compounds.

S.No.	Compound ID	Canonical SMILES	Family
1	ZINC95477953	COc1cc(-c2c3oc4cc(O)ccc4c3nc3c2c(O)nn3C2CCCC2)cc(OC)c1OC	Pyridine–pyrazole derivative
2	ZINC79497869	CC(C)n1nc(O)c2c(-c3ccc(OCC(N)=O)cc3)c3oc4cc(O)ccc4c3nc21	Pyridine–pyrazole
3	ZINC95480156	COc1cc(-c2c3oc4cc(O)ccc4c3nc3c2c(O)nn3C2CCCC2)ccc1OCC(N)=O	Pyridine–pyrazole derivative
4	ZINC95476071	Oc1ccc2c(c1)oc1c(-c3ccc4c(c3)OCO4)c3c(O)nn(C4CCCC4)c3nc12	Pyridine–pyrazole
5	ZINC95478185	COc1ccc(-c2c3oc4cc(O)ccc4c3nc3c2c(O)nn3C2CCCC2)cc1OCC(N)=O	Pyridine–pyrazole
6	ZINC95473654	COc1cc(-c2c3oc4cc(O)ccc4c3nc3c2c(O)nn3C2CCCC2)cc(OC)c1O	Pyridine–pyrazole
7	ZINC95473725	Oc1ccc2c(c1)oc1c(-c3ccc(O)c(O)c3)c3c(O)nn(C4CCCC4)c3nc12	Pyridine–pyrazole
8	ZINC95475599	COc1cc(-c2c3oc4cc(O)ccc4c3nc3c2c(O)nn3C2CCCC2)cc2c1OCCO2	Pyridine–pyrazole
9	ZINC95475957	COc1ccc(-c2c3oc4cc(O)ccc4c3nc3c2c(O)nn3C2CCCC2)cc1O	Pyridine–pyrazole
10	ZINC08792368	COc1ccc(CCNC(=O)COc2ccc3c(c2)c2ccnc4c5ccccc5c(=O)n3c24)cc1OC	Quinoline–lactam derivative
11	ZINC60353723	CCn1c2ccccc2c2cc(NS(=O)(=O)c3cnc4c(c3)c(C)nn4C)ccc21	Pyrazolopyridine–carbazole
12	ZINC13135380	CCOc1ccc(CCNC(=O)COc2ccc3oc4ccccc4c3c2)cc1OCC	Dibenzofuran–amide derivative
13	ZINC06409441	COc1ccc2cc(CN(C[C@@H]3CCCO3)S(=O)(=O)c3c(C)ccc4nsnc34)c(=O)[nH]c2c1	Benzothiadiazole derivative
14	ZINC72066827	CCn1c2ccccc2c2cc(NS(=O)(=O)c3cnc4onc(C)c4c3)ccc21	Carbazole derivative
15	ZINC25540664	O=c1c2ccc3oc4c(S(=O)(=O)O)cc(S(=O)(=O)O)cc4c4ccc(c(=O)n1CCO)c2c34	Xanthene derivative

Table S3. Best Docking Results of the 15 Shortlisted Compounds Based on MM-GBSA Binding Energy, –CDOCKER Score, and AutoDock Vina Binding Affinity.

S. No.	Compound ID	MM-GBSA (kcal/mol)	−CDOCKER Score	AutoDoc Vina Binding Affinity (Kcal/mol)
1	ZINC95477953	-40.5652	48.0376	-7.8
2	ZINC79497869	-46.1086	49.2066	-8.0
3	ZINC95480156	-49.5301	50.0290	-8.0
4	ZINC95476071	-66.4474	49.0958	-8.1
5	ZINC95478185	-59.4316	50.2211	-8.1
6	ZINC95473654	-121.867	54.6032	-8.7
7	ZINC95473725	-102.472	50.8604	-8.6
8	ZINC95475599	-38.7958	45.4561	-8.2
9	ZINC95475957	-41.8603	44.6012	-8.1
10	ZINC08792368	-97.7334	52.7331	-8.2
11	ZINC60353723	*-66.87*	40.8333	-8.2
12	ZINC13135380	*-54.99*	33.3168	-7.7
13	ZINC06409441	-49.9932	43.7788	-8.0
14	ZINC72066827	-66.11	48.8224	-8.1
15	ZINC25540664	-57.73	52.7303	-7.5

Table S4: Comparison of Experimentally Validated SARS-CoV-2 MPro Inhibitors and the Computationally Identified Lead Compounds (Lig-1, Lig-2 and Lig-3), Highlighting Key Binding Residues and Experimental Status.

S. No.	Compound	Experimental Status	Key Interacting Residues of MPro	Reference
1	Nirmatrelvir (PF-07321332)	In vitro, in vivo, Clinical	HIS41, CYS145, HIS163, GLU166, GLN189, MET165	Owen, D.R. et al. Science, 2021, 374, 1586–1593.
2	GC376	In vitro	His41, Cys145, His163, Glu166, Met165, Gln189	Vuong, W. et al. Nat. Commun., 2020, 11, 4282.
3	Baicalein	In vitro	Leu141, Gly143, Ser144, Cys145, His163, Glu166	Su, H. et al. Cell Research, 2020, 30, 678–692.
4	Ensitrelvir (S-217622)	In vitro, Clinical	His41, Cys145, Met165, Glu166, Gln189	Unoh, Y. et al. J. Med. Chem., 2022, 65, 6499–6512.
5	Boceprevir	In vitro	His41, Cys145, His164, Glu166, Gln189	Ma, C. et al. Cell Research, 2020, 30, 678–692.
6	Lig-1	Computational (This study)	HIS41, MET49, PHE140, LEU141, CYS145, SER144, ASN142, HIS163, and GLU166.	-

7	Lig-2	Computational (This study)	THR26, HIS41, MET49, PHE140, LEU141, CYS145, MET165	-
8	Lig-3	Computational (This study)	THR26, PHE140, GLU166, CYS145, HIS41, MET49, MET165, and LEU141	-

Validation of top pharmacophore hypotheses showing performance metrics including sensitivity, specificity, and area under the curve (AUC). Pharmacophore_01 exhibited the best predictive performance with an AUC of 0.781.

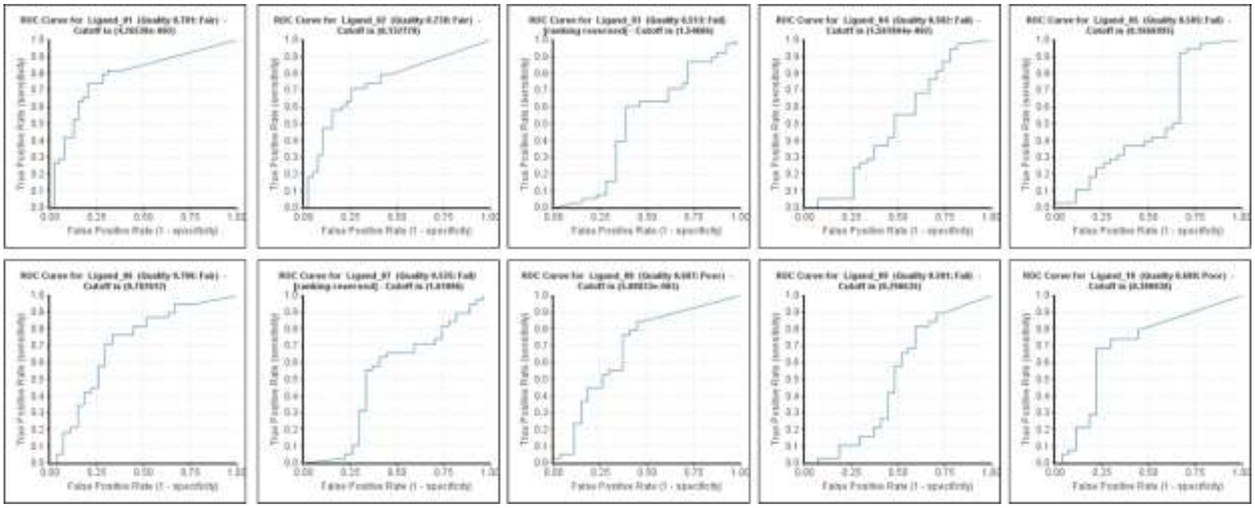

Figure S1. Receiver operating characteristic (ROC) curves of ten selected ligands (Ligand_01–Ligand_10) showing their classification performance. The x-axis represents the false positive rate (1 − specificity) and the y-axis represents the true positive rate (sensitivity). The area under the curve (AUC) and cutoff values are indicated for each ligand, reflecting their predictive accuracy. Among them, Ligand_01 demonstrated comparatively better ROC performance and AUC and was therefore selected for further evaluation.

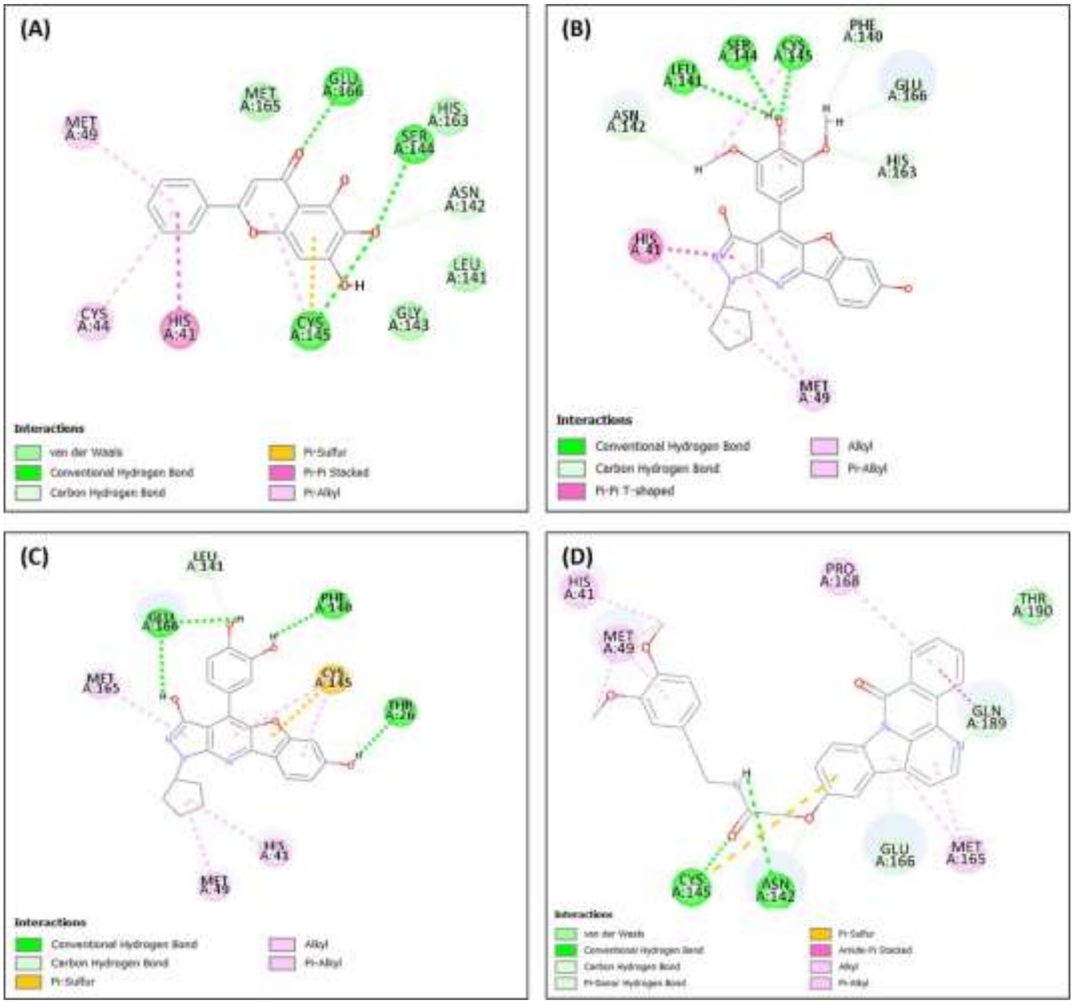

Figure S2. Two-dimensional (2D) protein–ligand interaction diagrams of the standard and selected ZINC compounds within the active site of the target protein. (A) Standard (Reference) ligand Baicalein, (B) ZINC95473654, (C) ZINC95473725, and (D) ZINC08792368. The diagrams illustrate key interactions including conventional hydrogen bonds, carbon hydrogen bonds, π–π stacking, π–alkyl, π–sulfur, and van der Waals interactions with surrounding amino acid residues. These interactions contribute to the stability and binding affinity of the ligands within the binding pocket of Mpro.
